# UK case control study of brain tumours in children, teenagers and young adults: a pilot study

**DOI:** 10.1186/1756-0500-7-14

**Published:** 2014-01-08

**Authors:** Richard G Feltbower, Sarah J Fleming, Susan V Picton, Robert D Alston, Diana Morgan, Janice Achilles, Patricia A McKinney, Jillian M Birch

**Affiliations:** 1 Division of Epidemiology and Biostatistics, School of Medicine, University of Leeds, Leeds LS2 9JT, UK; 2 Paediatric Haematology and Oncology, The General Infirmary at Leeds, Martin Wing, Leeds Teaching Hospitals NHS Trust, Leeds LS1 3EX, UK; 3 Cancer Research UK Paediatric and Familial Cancer Research Group, Manchester Academic Health Science Centre, University of Manchester, Manchester, UK

**Keywords:** Brain tumour, Children, Adolescent, Case–control study, Aetiology

## Abstract

**Background:**

Tumours of the central nervous system are the second most common group of childhood cancers in 0–14 year olds (24% of total cancers) and represent a major diagnostic group in 15–24 year olds. The pilot case–control study aimed to establish methodologies for a future comprehensive aetiological investigation among children and young adults.

**Methods:**

Eligible cases were newly diagnosed with an intracranial tumour of neuroepithelial tissue aged 0–24 years. The pilot recruited patients through Leeds and Manchester Principal Treatment Centres. Controls were drawn from general practice lists. Controls were frequency matched by age and gender.

**Results:**

We interviewed 49 cases and 78 controls comprising 85% of the target sample size. Response rates were 52% for cases and 32% for controls. Completion of the questionnaire was successful, with a very small proportion of missing data being reported (5-10%). The age distribution of cases and controls was similar with around three-quarters of interviewed subjects aged 0–14. Half of cases and almost two-thirds of controls reported using a mobile phone with the majority starting between 10–14 years of age. Prevalence of breastfeeding was lower in cases than controls (Odds Ratio 0.4; 95% CI 0.2-1.2), whilst cases were more likely to be delivered by caesarean section (OR 1.6; 95% CI 0.6-4.4). Cases were significantly more likely to have a birthweight > 3.5 kg compared to controls. Cases were also more likely to come from a family with 3 or more siblings than controls (OR 3.0; 95% CI 0.7-13.6). The majority of participants (>80%) were in favour of taking either blood or saliva to aid molecular epidemiological research.

**Conclusions:**

Successful methods were established for identifying and recruiting a high proportion of case subjects, exploiting strong links with the clinical teams at the treatment centres. Control procedures proved more difficult to implement. However, working closely with national clinical and professional research networks will enable improved control identification and recruitment, with good prospects for collecting biological samples in the future.

## Background

Tumours of the central nervous system (CNS) are the second most common group of childhood cancers comprising a quarter of all malignancies in patients aged 0–14 years with approximately 350 children diagnosed each year in the UK [1]. CNS tumours also represent a major diagnostic group in teenagers and young adults aged 15–24 years with around 150 new cases per year diagnosed in the UK representing 10% of all cancers in this age range [2]. Survival rates for CNS tumours in young people are generally poor when compared to other cancers occurring among 0–24 year olds: around 50% of these patients die from their disease and those who survive are at particular risk of severely debilitating late effects [3].

CNS tumours presenting in the young differ notably from those in older adults in terms of the cellular origins, pathological subtypes and anatomic site. The most common subtypes in young people are astrocytic tumours (50%) and embryonal tumours including medulloblastoma (25%) [4]. Apart from an increasing number of cancer predisposition syndromes associated with early onset CNS tumours, the causes of CNS tumours in children and young adults remain largely unknown.

The only established environmental risk factor for CNS tumours is ionising radiation [5-8]. Exposure to N-nitroso compounds through consumption of cured meat during pregnancy has been consistently reported as an aetiological factor [6,7]. Childhood CNS tumours have also been linked to residential pesticide exposure, traffic pollution, and parental occupations [8-11]. There is accumulating evidence of links between infections and CNS tumours particularly in the young. Supportive evidence for the involvement of infections comes from analyses of space-time clustering of incident cases, geographical and demographic variations in incidence and population mixing [12-16].

Pre-school nurseries have a high prevalence and diversity of infectious disease (e.g. [17]) and attendance can be considered a proxy for early exposure to infections. Exposure to infection in early life has been investigated in the context of a potential infectious aetiology for childhood type CNS tumours although findings have been inconsistent, varying according to tumour type and exposure of interest [18,19]. Atopic diseases, such as asthma, eczema and allergies, can be markers of immune dysfunction. There is evidence to suggest that atopic conditions may confer a reduced risk of CNS tumours in children [20,21]. Furthermore, specific HLA alleles and haplotypes are associated with relatively higher or lower risks of childhood ALL and possibly also CNS tumours [22,23]. Higher birthweight, especially those born weighing over 4000 g, has also been implicated as a possible causal risk factor for childhood brain tumours [24], whilst a protective association has been described for maternal farm residence during pregnancy and postnatal contact with birds [25].

As a forerunner to a population-based case control study of neuroepithelial CNS tumours in children, teenagers and young adults we aimed to undertake a pilot study involving a multidisciplinary team comprising paediatric and adolescent oncologists, research nurses, and epidemiologists. The aims were to 1) establish procedures for optimal case and control ascertainment, 2) pilot a questionnaire and study materials, 3) optimise the collection and storage of biological samples 4) develop a protocol and grant application for the full study.

## Methods

### Case–control selection

Eligible cases were those children and young people who were aged 0–24 years at diagnosis presenting with a primary intracranial tumour of neuroepithelial tissue as defined by WHO classification [4]. Tumours were classified into the following subtypes: ependymoma, astrocytoma, embryonal and other specified tumours. Cases were identified through clinical teams based in the two UK Principal Treatment Centres of Leeds and Manchester, comprising dedicated paediatric and Teenage and Young Adult oncology units. Approaches to patients/parents were made at a time recommended by the clinical teams. Written, informed consent to take part in the pilot study was obtained. Response rates were assessed by age group, gender, CNS subtype and centre.

Controls from the Leeds centre were randomly selected from general practice (GP) lists to identify a population-based sample and provide access to medical records. As part of the feasibility process, controls were frequency matched according to the age (0–24 years) and sex distribution of the case sample. GP practices were selected whose population demographic (age, sex, social class and population density) reflected those of the larger geographical area. Once approval was obtained, a study administrator based themselves in the practice and randomly selected a list of eligible participants. Study invitation letters were distributed on behalf of the person’s GP. Where a control refused to take part, replacement controls were used and the socio-demographic breakdown of response rates monitored to assess the representation of the participants. From the Manchester centre, three friend controls who fulfilled the required age and sex were selected and interviewed. Numerous GP practices were approached but despite extensive efforts and involvement with the Primary Care Research Network (PCRN), a group which supports clinical research studies involving primary care services in England, we were unable to recruit any practices (see Results).

The pilot study set out to recruit and interview 25 cases and 50 controls per centre (50 cases and 100 controls in total).

### Interview materials and processes

Exposure prevalence was assessed through information collected from face-to-face interviews. The interview proforma was designed to be compatible with a large parallel international case–control study covering the Nordic countries [26], a copy of which is provided in the Additional file 1. For each centre, an experienced research nurse co-ordinated and conducted interviews with participants and their families. Information was collected on the health of the young person, parental health, the index person’s early social habits as a child and the family histories of cancer for cases. Information was captured by a trained interviewer who administered either a Computer Assisted Personal Interview (CAPI) questionnaire adapted from a parallel Nordic study [26], or a paper-based questionnaire which was then transferred onto a Microsoft Access database. Parental interviews were undertaken for those aged under 12 years; for older subjects, both parents and cases were interviewed (Additional file 1).

Ethical approval for the study was granted by the North West Research Ethics Committee in July 2007 (reference number 07/MRE08/46) and informed consent obtained for every participant. The study conformed to the principles embodied in the Declaration of Helsinki. The recruitment periods were September 2007 to March 2009 in Leeds and June 2008 to June 2010 in Manchester.

### Statistical analysis

Conditional logistic regression stratified by age (5-year age groups) and sex was undertaken to derive odds ratios (OR) and 95% CI. Adjustment for deprivation was carried out using the Townsend score of the child’s address at diagnosis by the use of Townsend score quintiles based on the UK population distribution. In view of the limited sample size and power and range of possible aetiological factors involved in the development of CNS tumours in children and young people, we undertook a careful regression analysis including a small number of risk factors which were deemed important based on the epidemiological literature (breastfeeding, caesarean section, birthweight, number of siblings, mobile phone usage, contact with animals). A full list of risk factors collected from the interviews is provided in the Additional file 1. All analyses were carried out using Stata version 12.1.

## Results

### Recruitment and participant characteristics

**Figure 1:**
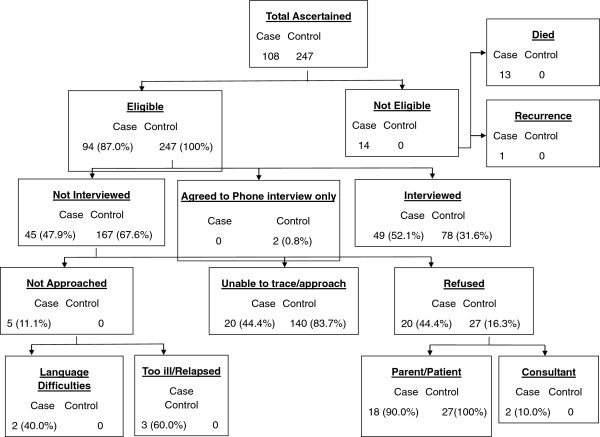
Recruitment flowchart (all centres combined).

Aggregating the data from across both centres yielded 49 cases and 78 controls who were interviewed. Overall, although both centres experienced some problems in terms of recruitment, across both centres we recruited 85% of the target sample size. The flowchart in Figure 1 describes the recruitment pathways and number of subjects identified at each stage for both centres combined.

Table 1 summarises the age, sex and CNS subtype distribution by case and control status. 81% of cases were aged 0–14 years at interview compared to two-thirds of controls. There was a notable excess of controls diagnosed aged 15–19 (22%) compared to cases (10%). Slightly more cases were male (59%) with a slightly higher percentage of controls being female (53%).

**Table 1:** Demographic characteristics of the study participants according to case (n = 49) and control (n = 78) status

	**Cases**		**Controls**	
	**n**	**%**	**n**	**%**
**Age**				
0-4	16	32.7	19	24.4
5-9	13	26.5	16	20.5
10-14	11	22.5	17	21.8
15-19	5	10.2	17	21.8
20-24	4	8.2	9	11.5
**Sex**				
Male	29	59.3	34	46.8
Female	20	40.7	44	53.2
**Study centre**				
Leeds	18	36.7	76	97.4
Manchester	31	63.3	2	2.6
**Diagnosis of cases (ICCC grouping)**				
Ependymomas	8	16.3		
Astrocytomas	27	55.1		
Embryonal	11	22.5		
Other specified	3	6.1		

Response rates were 52% and 32% for cases and controls respectively out of those who were eligible for the study (Figure 1). We found recruitment to be a significant challenge notably for cases in Leeds and controls in Manchester. These were largely attributable to changes in NHS governance and the GP contract during the recruitment phase. Main reasons for not taking part were reported as refusal (4 cases and 27 controls from Leeds; 9 cases from Manchester) and untraceable subjects (5 cases and 139 controls from Leeds; 15 cases from Manchester). Completion of the questionnaire however was a success, with a very small proportion of missing data being reported (typically 5-10% for each variable). Where missing data were present, this largely related to the same individuals.

From Manchester deprivation scores were available from cases who did not take part and it was found that there was no significant difference in Townsend score between the two groups. In the Leeds area deprivation was available for interviewed and non-interviewed controls and when comparing deprivation quintile there was found to be a significant trend of reducing participation with increasing quintiles of deprivation (OR: 0.6, 95% CI: 0.5-0.8, p < 0.001).

Half of cases and almost two-thirds of controls indicated that they had used a mobile phone (Table 2). The majority of respondents who used a mobile device began doing so when they were 10–14 years of age. Excluding missing data, the prevalence of breastfeeding was lower in cases (73%) compared to controls (83%), whilst cases (25%) were more likely to be delivered by caesarean section than controls (15%) (Table 2). 61% of cases had a birthweight in excess of 3.5 kg compared to only 36% of controls. Cases were also more likely to come from a family with 3 or more siblings (31%) than controls (12%). A lower proportion of cases (29%) reported having regular contact with animals outside than controls (40%) (Table 2).

**Table 2:** Results of the Logistic Regression modelling according to selected risk factors

	**Cases**		**Controls**		Odds ratio*	**95% confidence interval**	
	**n**	***%***	**n**	***%***			
**Ever breastfed?**							
No	12	*24.5*	13	*16.7*	REF		
Yes	32	*65.3*	62	*79.5*	0.4	0.2	1.2
Missing	5	*10.2*	3	*3.9*			
**Caesarean section?**							
No	33	*67.4*	63	*80.8*	REF		
Yes	11	*22.5*	11	*14.1*	1.6	0.6	4.4
Missing	5	*10.2*	4	*5.1*			
**Birthweight (g)**							
<2500	2	*4.1*	3	*3.9*	1.5	0.2	11.9
2500-2999	4	*8.2*	12	*15.4*	1.0	0.2	4.0
3000-3499	11	*22.5*	30	*38.5*	REF		
3500-3999	16	*32.7*	16	*20.5*	2.9	1.0	8.2
4000+	11	*22.5*	9	*11.5*	3.7	1.1	12.4
Missing	5	*10.2*	8	*10.3*			
**Number of siblings**							
0	5	*10.2*	8	*10.3*	REF		
1	20	*40.8*	37	*47.4*	1.1	0.3	4.3
2	9	*18.4*	21	*26.9*	1.0	0.2	4.3
3+	15	*30.6*	9	*11.5*	3.0	0.7	13.6
Missing	0	*0.0*	3	*3.9*			
**Spoken on a mobile phone more than 20 times?**							
No	23	*46.9*	28	*35.9*	REF		
Yes	26	*53.1*	49	*62.8*	0.9	0.2	3.3
Missing	0	*0.0*	1	*1.3*			
**Any animals kept at home?**							
No	11	*22.5*	14	*17.9*	REF		
Yes	38	*77.5*	62	*79.5*	1.0	0.3	2.9
Missing	0	*0.0*	2	*2.6*			
**Regular contact with animals outside the home?**							
No	35	*71.4*	47	*60.3*	REF		
Yes	14	*28.6*	31	*39.7*	0.7	0.3	1.5
Missing	0	*0.0*	0	*0.0*			

*Odds ratios calculated using conditional logistic regression stratified by age (5-year groups) and sex, analyses were adjusted for deprivation by the use of Townsend score quintiles based on the UK population distribution.

*REF*, Reference category.

Logistic regression modelling for selected birth related and environmental factors (Table 2) showed some evidence of a reduced risk for ever having being breastfed (OR: 0.4, 95% CI: 0.2-1.2). There was a statistically significant finding of increased risk with increased birth weight compared to normal weight (3500-3999 g OR: 2.9, 95% CI: 1.0-8.2, ≥4000 g, OR: 3.7, 95% CI: 1.1-12.4). Other factors explored did not appear to have an association with brain tumour risk.

Of the 12 cases and 25 controls who responded about their willingness to provide a blood or saliva sample to carry out future biological research, all cases and controls said they would agree to provide saliva whilst 89% of cases and 81% of controls would agree to provide a blood sample.

## Discussion

Through this pilot study, we have demonstrated that by working closely as a multidisciplinary team, recruitment of participants diagnosed with brain tumours is feasible as part of a ‘case-control’ design to address aetiological questions, despite the huge challenges facing these young people shortly after diagnosis.

In terms of addressing the aims of the study, we developed successful methods for identifying and recruiting a high proportion of case subjects by exploiting our strong links with local clinicians and research nurses. Control procedures proved more difficult; nonetheless, this pilot study was informative and we propose the following recommendations to facilitate the design and recruitment of future UK case–control investigations involving childhood and young adult cancer:

1. Close collaboration with primary care and the National Institute for Health Research (NIHR) Comprehensive Clinical Research Network (CCRN), a body which oversees all clinical NHS-based research in England and which supports widening research participation to improve patient benefit across all clinical domains. This will help to optimise recruitment for both cases and controls.

2. Engagement with the PCRN and National Cancer Research Institute (NCRI) Primary Care Clinical Studies Group (CSG) to identify general practices which are familiar with research studies, the latter a professional group helping to develop major primary care oncology research studies in the UK.

3. Collaboration with the relevant Childhood Cancer and Leukaemia Group (CCLG) sub-group, e.g. the CNS sub-group, a national group of professionals dedicated to improving the delivery of care for young people with cancer.

4. Collection of saliva samples for the purposes of molecular or genetic epidemiology.

Both Leeds and Manchester experienced a number of problems relating to recruitment of controls via GP practices. In Leeds, procedures for identifying controls were resource intensive leading to a much lower than anticipated recruitment rate of 31%. The delayed start in Manchester meant adhering to new NHS structures and despite full ethical and Research and Development/Caldicott Guardian approval for the control recruitment protocol and acceptance of the study onto the NIHR/PCRN portfolio, little progress was made in identifying GP practices willing to participate in the research. Exhaustive efforts over a long period of time were made by the Manchester staff to engage with general practices both through the PCRN and directly to practices with little success.

We believe that the new NHS General Medical Services contract for General Practice, which was implemented in 2004 and allocated certain resources to GPs based on how well they manage patient care (the Quality and Outcomes Framework), may have influenced the willingness of GP practices throughout Manchester to participate. We have since taken advice from the national NCRI Primary Care CSG to help develop control recruitment procedures for future research by ensuring that we work closely with the regional CCRN. We are also exploring alternative sources of control subjects such as child health records through our existing links with primary care.

Completion of the questionnaire was a success, with a very small proportion of missing data being reported. The collection of biological samples would be an integral part of future epidemiological research in this field. We explored the possibility of exploiting the national CCLG tumour bank samples in conjunction with case control research projects. The Brain Tumour sub-group of the CCLG was fully supportive and indicated willingness to collaborate with future studies. All tumour and blood samples collected by the CCLG adhere to specific protocols which would be closely mirrored in future studies. Although we did not collect biological material, we did however ask participants about their willingness to provide biological samples and there was a clear consensus in favour of taking either blood or saliva to aid molecular epidemiological research. Participants also stated that saliva samples would be more readily donated than blood, particularly from younger controls.

Although our pilot study had limited statistical power, findings agreed with previous aetiological work in showing an increased risk associated with birthweight greater than 3500 g [24]. However, our reported non-significant protective association with breastfeeding is contrary to previous findings [27,28] and may have been due to chance. The use of friend controls may have led to a degree of overmatching, although the number of participants selected in this manner (n = 3) is unlikely to have had a major effect on the parameter estimates reported in Table 2. Control participation in Leeds was also related to deprivation, with higher levels of participation from more affluent areas. This potential participation bias may have contributed to the higher observed rates of breastfeeding, smaller sibling size and mobile phone usage in the control sample. Nevertheless, as we reported the results for a selected range of risk factors from a relatively small feasibility study, odds ratios should be interpreted with due caution and not be taken as evidence or absence of any causal association.

Feedback from participants has provided us with key information with which to revise the study questionnaires and recruitment procedures to ensure that participation rates in future case–control studies can be maximised. It has provided a valuable insight into questionnaire design and recruitment procedures, particularly in terms of overcoming problems associated with the identification of suitable healthy control subjects.

## Conclusions

In summary, this pilot has provided us with all the elements necessary to produce a full protocol for a future UK study, including extensive documentation on all aspects of recruiting and approaching case and control subjects and their families. Findings from this pilot will provide essential information for refining the methods for a future large, multi-centre case–control study.

## Consent

Written informed consent was obtained from the patient or their guardian/parent/next of kin for the publication of this report.

## Competing interests

The authors declare that they have no competing interest.

## Authors’ contributions

JB and PM devised the study; DM and JA organised the interviews and gathered the data for the study from participants; SF and RF carried out the statistical analysis; RF drafted the manuscript; all authors provided critical comments and approved the contents of the paper prior to submission. All authors read and approved the final manuscript.

## Supplementary Material

Additional file 1 Family questionnaire. Click here for file
