# Supplementary material for: UK case control study of brain tumours in children, teenagers and young adults: a pilot study
Source: BMC Res Notes. 2014 Jan 8;7:14. doi: 10.1186/1756-0500-7-14 (PMC3922821; doi:10.1186/1756-0500-7-14)
Supplement: Additional file 1 — Family questionnaire. [file 1756-0500-7-14-S1.pdf]

CEFALO
International Case Control Study
of Childhood Brain Tumour

Family Questionnaire

Including questions within the following subjects:

Introduction Questions
The Child's use of Mobile Phones
The Child's Contact with Animals
X-rays and Scanning
The Child's Health History
Milestones in the Child's Development
The Child's Contact with other Children
The Child's Family's Health
The Pregnancy and the Birth of the Child
Parental Background

(Please note: Do not read answers "Do not remember/ do not know" and "No answer" aloud.)

Introduction questions

Whole screen text

INTRODUCTION:

Thank you for agreeing to meet with me today. As you recall, we are studying brain tumour in children and adolescents from the age of seven to nineteen. We hope to learn more about the possible causes of childhood brain tumour. We will compare responses from families with a child who has brain tumour to responses from families with a child who does not.

Please give as much information as you can about each question, as well as any other information about yourself or your child's background that you think would be useful. Note that we are interested in the entire life of the child, that means from birth.

I will read all questions and answer alternatives aloud to you. Then I would like you to state the alternative that fits the best. In some questions it is possible to tick more than one answer. At the end of the interview you will have the opportunity to leave your own comments.

Your participation in this study is voluntary. You may choose not to answer any question, or to end the interview at any time. (Cases only: This will not affect any treatment or care your child receives.)

[General note for the interviewer: In many of the questions it is possible to indicate age or date very precisely. If the interviewee does not remember the exact age or date, ask him/her to make a guess or indicate just the year. In the latter case, leave the form for the months blank.]

The first few sections of the interview concern [child]'s use of mobile phones, contact with animals, health and allergies. I would like [child] to help by answering these initial questions, if he/she wants. The remainder of the interview is mainly directed to the mother.

Study no.

|__|__|__|__|__|__|

Q 1 Name and code of interviewer

|__|__| (code), name: ______________________________________

Q 2 Country where interview is conducted

1. Denmark
2. Sweden
3. Norway
4. Switzerland

Q 3 What is the first name of the child?

Name: ____

Q 4 End of exposure date (always 00 in day)

|__|__| (day) |__|__| (month) 200|__| (year)

Q 5 What is [child]`s birthday ?

|__|__| (day) |__|__| (month) |__|__|__|__| | (year)

Q 6 Is it a case or control?

☐ Case
☐ Control

Q 7 Biological mother?

☐ Yes
☐ No
☐(Do not remember/ do not know)
☐ (no answer)

Q 8 Date of interview:

|__|__| (day) |__|__| (month) 200|__| (year)

Q 9 Start of interview:

_ _ : _ _ (hours, minutes)

Q 10 Where is the interview taking place?

☐ Home
☐ Hospital/doctor’s
☐ Other, specify ____

Q 11 Who is present at the interview: (Tick more boxes if appropriate)

☐ Child
☐ Mother
☐ Father
☐ Other (relation to child): ______________________________________

Questions regarding child's mobile phone(s)

Flow
I now want to ask you about [child]'s use of mobile phones - when [child] calls someone and when someone calls [child]. In the following questions we only want you to mention what happened before [exposure date/month].

Q 12 **Has [child] *spoken* on a mobile phone more than 20 times during his/her life?**
Please note that I am interested in hearing of *any* mobile phone that the child might have used.

- ☐ Yes
- ☐ No → *please go to* **Q 38**
- ☐ (Do not remember/ do not know → *please go to* **Q 38**)
- ☐ (No answer → *please go to* **Q 38**)

Q 13 **How old was [child] when he/she first started <u>talking</u> on a mobile phone?**

- ☐ Age: |__|__| years and |__|__| months

or

- ☐ Date: |__|__| (month) |__|__|__|__| (year)
- ☐ (Do not remember/ do not know)
- ☐ (no answer)

Q 14 **Has [child] ever had a mobile phone on his/her own?**
Also if the subscription was in somebody else's name, but the child was the main user of this subscription.

- ☐ Yes→ *please go to calendar for registration*
- ☐ No → *please go to* Q 15*)*
- ☐ (Do not remember/ do not know → *please go to* Q 15*)*
- ☐ (No answer → *please go to* Q 15)

Q 15 <u>**How often does/did [child] talk on somebody else's mobile phone?** (Tick only one)</u>

- ☐ Less than once a <u>month</u>→ *please go* Q 30
- ☐ Some times a month → *please go* Q 30
- ☐ 1-2 times per <u>week</u>→ *please go to* Q 16
- ☐ 3-6 times per <u>week</u>→ *please go to* Q 16
- ☐ 1-2 times a <u>day</u>→ *please go to* Q 16
- ☐ 3-4 times a <u>day</u>→ *please go to* Q 16
- ☐ 5 times or more a <u>day</u> → *please go to* Q 16
- ☐ (Do not remember/ do not know) → *please go* Q 30
- ☐ (No answer) → please go Q 30

Q 16 When [child] talks/talked on somebody else's mobile phone, which phrase fits/fitted his/her usage the best? No distinction between calls made by or received by the child. (Tick only one)

When I am talking on somebody else's mobile phone, I mostly talk...

☐ a little, i.e. short messages (about 1 min) → *please go to* Q 30
☐ 3 mins → *please go to* Q 30
☐ 6 mins → *please go to* Q 30
☐ a bit longer, about 10 mins or more → *please go to* Q 30
☐ (Do not remember/ do not know → *please go to* Q 30)
☐ (No answer → *please go to* Q 30)

Whole screen
Now we will help each other to fill out a table of the different subscriptions mainly used by [child] – whether the different mobile phones he/she has used have belonged to [child] him/herself, or somebody else.

(NOW LEAVE THE LAP-TOP AND FILL IN THE FOLLOWING PAPER SHEETS BY HAND) (THE CALENDAR AND THE QUESTIONS CONCERNING AMOUNT OF USE).

(Introduce paper calendar)
In the following questions we only want you to mention what happened before [exposure date/month].

When did [child] have his/her first mobile phone?
(Each subscription gets a number.
Each period gets a letter and the number of the related subscription as a prefix.)
(Ask for each subscription;)

For which period did you have this subscription?
(If the subscription lasts for more than 2 years then ask:)

During the period you had this subscription, did your talking habits change?
(If yes, then subdivide the subscription period into periods of at least 1 year. If there was a continuous increase/decrease in the talking activity, then mark a start period and an end period.)

Which operator?

Was it NMT /GSM /3G?

What type of subscription was it?

1. Phone number: Firstly, I would like to register the different phone numbers of the different subscriptions. (Register in calendar)
(Suggestions to where the family can look for the child's previous mobile phone numbers: checking in old phone books (or newer ones, which might have the old number indicated, even if this is crossed out), ask family members to check whether they still have the old number saved in the address book of their mobile phone. Otherwise the family might still have old bills where the number is indicated. It is important for us to get the right number in order to be able to obtain traffic data from the mobile phone operators.)

2. Name of subscriber: I would also like to know in what name the different subscriptions were registered/established (as it is not possible to register for a subscription when under 18, the subscriptions are normally established in the name of a parent). (Register in calendar)

3. First user: I would also like to know whether the subscriptions in question had been used by others before the child took over the phone and/or subscription or whether the subscription was established particularly for the child. (Register in calendar)

4. Last user: I would like to know whether the subscriptions in question had been used by others after the child stopped using the subscription. (Register in calendar)

(Fill in the following annexes (see separate lists):
- Annex 1 – List of Different Subscriptions used by Child
- Annex 2 – Mobile Phone Usage)

(When the calendar and usage questions have been filled out, proceed to the following computerised questions.)

Flow
I would now like to ask you whether [child] used any other mobile phones before having one of his/her own (the first one marked in calendar).

Q 17 **Prior to the first subscription we marked in the calendar, did [child] use a mobile phone for talking more than 20 times?**
e.g. using parents' or siblings' mobile phone

☐ Yes
☐ No → *please go to* **Q 19**
☐ (Do not remember/ do not know → *please go to* **Q 19**)
☐ (No answer → *please go to* **Q 19**)

Q 18 **Prior to the first subscription we marked in the calendar, how often did [child] <u>talk</u> on a mobile phone?** (Tick only one)

☐ Less than once a <u>month</u>
☐ Some times a month
☐ 1-2 times per <u>week</u>→ *please go back to Appendix 2 for registration*
☐ 3-6 times per <u>week</u>→ *please go back to Appendix 2 for registration*
☐ 1-2 times a <u>day</u>→ *please go back to Appendix 2 for registration*
☐ 3-4 times a <u>day</u>→ *please go back to Appendix 2 for registration*
☐ 5 times or more a <u>day</u> → *please go back to Appendix 2 for registration*
☐ (Do not remember/ do not know)
☐ (No answer)

Q 19 **When you look at the calendar, did [child] during any of the mentioned subscriptions/periods <u>regularly</u> use other mobile phones?**
e.g. using parents', siblings' or friends' mobile phone
In terms of regularly I mean once or more per week.

☐ Yes
☐ No → *please go to* **Q 24**
☐ (Do not remember/ do not know → *please go to* **Q 24**)
☐ (No answer → *please go to* **Q 24**)

Q 20	**In relation to which subscription/period?**
	1st subscription/period |__|__|
	2nd subscription/period |__|__|
	3rd subscription/period |__|__|
	4th subscription/period |__|__|
	☐ (Do not remember/ do not know → *please go to* **Q 24**)
	☐ (no answer → *please go to* **Q 24**)

Flow
The following questions (Q 21 - Q 23) will be repeated for each subscription or period.

Q 21 How often does/did [child] talk on somebody else's mobile phone? (Tick only one)

1st	2nd	3rd	4th	
☐	☐	☐	☐	1-2 times per week
☐	☐	☐	☐	3-6 times per week
☐	☐	☐	☐	1-2 times a day
☐	☐	☐	☐	3-4 times a day
☐	☐	☐	☐	5 times or more a day
☐	☐	☐	☐	(Do not remember/ do not know)
☐	☐	☐	☐	(No answer)

Q 22 When [child] talks/talked on somebody else's mobile phone, which phrase fits/fitted his/her usage the best? No distinction between calls made by or received by the child. (Tick only one)

When I am talking on somebody else's mobile phone, I mostly talk ….

1st	2nd	3rd	4th	
☐	☐	☐	☐	a little, i.e. short messages (about 1 min)
☐	☐	☐	☐	3 mins
☐	☐	☐	☐	6 mins
☐	☐	☐	☐	a bit longer, about 10 mins or more
☐	☐	☐	☐	(Do not remember/ do not know)
☐	☐	☐	☐	(No answer)

Q 23 Did it happen in relation to other subscriptions/periods?

1st	2nd	3rd	4th	
☐	☐	☐	☐	Yes → *please go to* **Q 20**
☐	☐	☐	☐	No
☐	☐	☐	☐	(Do not remember/ do not know)
☐	☐	☐	☐	(No answer)

(End: Repeated questions Q 21 - Q 23 for each subscription or period)

Whole screen
I would like to ask for your permission to gain access to the exact use of mobile phone via the registries of the telephone operators. This is essential to get as good an impression of [child]'s

phone use as possible. The information we will gather from the operators solely concerns the duration and frequency of calls. We will thus <u>not</u> gather information on <u>which</u> numbers were called or the like.

Q 24 Would you give us permission to gain access to [child]'s use of mobile phones via the registries of the mobile phone operators?

- ☐ Yes
- ☐ No → *please go to* **Q 30**
- ☐ (Do not remember/ do not know → *please go to* Q 30)
- ☐ (No answer → *please go to* Q 30)

Flow

Finally, I would like to know, for each subscription, whether [child] was the sole user of the mobile phone.

Q 25 When you look at the calendar, did [child] during any of the mentioned subscriptions/periods <u>regularly</u> lend out his/her mobile phone?
In terms of regularly I mean once or more per week.

- ☐ Yes
- ☐ No → *please go to* **Q 30**
- ☐ (Do not remember/ do not know → *please go to* **Q 30**)
- ☐ (No answer → *please go to* **Q 30**)

Q 26 In relation to which subscription/period?

1st subscription/period |__|__|

2nd subscription/period |__|__|

3rd subscription/period |__|__|

4th subscription/period |__|__|

- ☐ (Do not remember/ do not know → *please go to* **Q 30**)
- ☐ (No answer → *please go to* **Q 30**)

Q 27 How often does/did somebody else talk on [child]'s mobile phone? (Tick only one)

1st	2nd	3rd	4th	
☐	☐	☐	☐	1-2 times per <u>week</u>
☐	☐	☐	☐	3-6 times per <u>week</u>
☐	☐	☐	☐	1-2 times a <u>day</u>
☐	☐	☐	☐	3-4 times a <u>day</u>
☐	☐	☐	☐	5 times or more a <u>day</u>
☐	☐	☐	☐	(Do not remember/ do not know)
☐	☐	☐	☐	(No answer)

Q 28 **When somebody else talks/talked on [child]'s mobile phone, which phrase fits/fitted his/her usage the best?** No distinction between calls made by or received. (Tick only one)

When somebody else talks on [child]'s mobile phone, he/she mostly talks ....

1st	2nd	3rd	4th	
☐	☐	☐	☐	a little, i.e. short messages (about 1 min)
☐	☐	☐	☐	3 mins
☐	☐	☐	☐	6 mins
☐	☐	☐	☐	a bit longer, about 10 mins or more
☐	☐	☐	☐	(Do not remember/ do not know)
☐	☐	☐	☐	(No answer)

Q 29 **Did it happen in relation to other subscriptions/periods?**

1st	2nd	3rd	4th	
☐	☐	☐	☐	Yes → *please go to* **Q 26**
☐	☐	☐	☐	No
☐	☐	☐	☐	(Do not remember/ do not know)
☐	☐	☐	☐	(No answer)

(Repeat for all subscriptions)

Q 30 **Who answered these questions?** (Tick only one)

☐ Child
☐ Mother
☐ Mother and child
☐ Father
☐ Father and child
☐ Only parents
☐ Together

General questions regarding child's habits of mobile phone use

Flow
I would now like to ask some general questions about [child]'s habits of mobile phone use.

Q 31 **When [child] speaks on a mobile phone, which ear does he/she mostly use, the right or the left ear??** (I am also interested in an answer even if [child] uses a handsfree kit.) (Tick only one)

☐ Right ear
☐ Left ear
☐ Both
☐ (Do not remember/ do not know → *please go to* **Q 33**)
☐ (No answer → *please go to* **Q 33**)

Q 32 Has it always been like this?

☐ Yes → *please go to* **Q 33**
☐ No
☐ (Do not remember/ do not know)
☐ (No answer)

When was it different?

☐ Age: before |__|__| years and |__|__| months
or
☐ Date: before |__|__| (month) |__|__|__|__| (year)
☐ (Do not remember/ do not know)
☐ (No answer)

Which ear did he/she use?

☐ Right ear
☐ Left ear
☐ Both
☐ (Do not remember/ do not know)
☐ (No answer)

Q 33 Is [child] right or left handed? (Tick only one)

☐ Right handed
☐ Left handed
☐ No preference
☐ (Do not remember/ do not know)
☐ (No answer)

Q 34 Has [child] ever used a handsfree, a headset with a microphone or the mobile's loudspeaker when speaking on his/her mobile phone?
[Note for the interviewer: i.e. the child does not hold the mobile against his/her ear]

☐ Yes
☐ No → *please go to* **Q 37**
☐ (Do not remember/ do not know → *please go to* **Q 37**)
☐ (No answer → *please go to* **Q 37**)

Q 35 Was it for a period of at least 6 months (consecutive months)?

☐ Yes
☐ No → *please go to* **Q 37**
☐ (Do not remember/ do not know → *please go to* Q 37)
☐ (No answer → *please go to* Q 37**)**

Q 36 For what periods of time did [child] use a handsfree, a headset or loudspeaker?

1st period:

☐ Child's age at the time:
|__|__| years and |__|__| month(s) until
|__|__| years and |__|__| month(s) (66 if still using)

or

☐ Date:
From |__|__| (month) |__|__|__|__| (year)
until |__|__| (month) |__|__|__|__| (year) (66 if still using)

☐ (Do not remember/ do not know)
☐ (No answer)

During this period, how often did [child] use a handsfree, a headset or loudspeaker while talking on the mobile phone?

☐ Sometimes
☐ Half the time
☐ Almost all the time
☐ (Do not remember/ do not know)
☐ (No answer)

Were there other periods when [child] used a handsfree, a headset or loudspeaker for at least 6 months?

☐ Yes
☐ No → *please go to* **Q 37**
☐ (Do not remember/ do not know → *please go to* **Q 37**)
☐ (No answer → *please go to* **Q 37**)

2nd period:

☐ Child's age at the time:
|__|__| years and |__|__| month(s) until
|__|__| years and |__|__| month(s) (66 if still using)

or

☐ Date:
From |__|__| (month) |__|__|__|__| (year)
until |__|__| (month) |__|__|__|__| (year) (66 if still using)

☐ (Do not remember/ do not know)
☐ (No answer)

During this period, how often did [child] use a handsfree, a headset or loudspeaker while talking on the mobile phone?

☐ Sometimes
☐ Half the time
☐ Almost all the time
☐ (Do not remember/ do not know)
☐ (No answer)

Q 37 Who answered these questions? (Tick only one)

- ☐ Child
- ☐ Mother
- ☐ Mother and child
- ☐ Father
- ☐ Father and child
- ☐ Only parents
- ☐ Together

Questions about the child's use of cordless phones

Flow
Note that in the following questions regarding cordless phones no distinction is made between when [child] calls somebody or when somebody calls [child].

In the following questions we only want you to mention what happened before [exposure date/month].

Q 38 Has [child] ever used a cordless phone in his/her home? (A fixed line telephone without a wire that you can carry with you throughout the house.)

- ☐ Yes
- ☐ No → *please go to* **Q 42**
- ☐ (Do not remember/ do not know → *please go to* **Q 42**)
- ☐ (No answer → *please go to* **Q 42**)

Q 39 Which period did [child] regularly use a cordless phone in his/her home? By regular we mean at least once a week or more during a longer period, i.e. half a year or more.

☐ Age: From |__|__| years and |__|__| months
To |__|__| years and |__|__| months

or

☐ Date: From |__|__| (month) |__|__|__|__| (year)
To |__|__| (month) |__|__|__|__| (year)

☐ (Do not remember/ do not know)

☐ (No answer)

Continuation 2

☐ Age: From |__|__| years and |__|__| months
To |__|__| years and |__|__| months

or

☐ Date: From |__|__| (month) |__|__|__|__| (year)
To |__|__| (month) |__|__|__|__| (year)

Continuation 3

☐ Age: From |__|__| years and |__|__| months
To |__|__| years and |__|__| months

or

☐ Date: From |__|__| (month) |__|__|__|__| (year)
To |__|__| (month) |__|__|__|__| (year)

Q 40 How often did [child] use a cordless phone in the first 3 years he/she used it regularly?
Now I am just interested in times when the child was actually speaking/listening on the phone and not just handing it over. (Tick only one)

☐ Less than once a week
☐ 1-2 times a week
☐ 3-6 times a week
☐ 1-2 times a day
☐ 3-4 times a day
☐ 5 times or more a day
☐ (Do not remember/ do not know)
☐ (No answer)

Q 41 **When [child] talked on the cordless phone during these three years, which phrase fitted the best?** (Tick only one box)

I mostly talked ….

- ☐ a little, i.e. short messages (about 1 min)
- ☐ 3 mins
- ☐ 6 mins
- ☐ a bit longer, about 10 mins or more
- ☐ (Do not remember/ do not know)
- ☐ (No answer)

Q 42 **Who answered these questions?** (Tick only one)

- ☐ Child
- ☐ Mother
- ☐ Mother and child
- ☐ Father
- ☐ Father and child
- ☐ Only parents
- ☐ Together

Questions regarding the child's contact with animals

Flow
I would now like to ask you about the animals that [child] has been in regular contact with during his/her childhood.

In the following questions we only want you to mention what happened before [exposure date/month].

Q 43 **After [child] was born, were any animals kept at his/her home?** We are interested in both indoor and outdoor animals.
[Note for the interviewer: Provide interviewees with a list of all the animals.]

- ☐ Yes
- ☐ No → *please go to* **Q 45**
- ☐ (Do not remember/ do not know → *please go to* **Q 45**)
- ☐ (No answer → *please go to* **Q 45**)

Q 44 What kind of animals/pets? (Show list of animals)
Indoor animal: an animal that is kept inside for most of the time, e.g. sleeps inside, for more than 6 months a year.
(Outdoor animal: an animal that is kept outside for most of the time, for more than 6 months a year.)

(If you tick both inside and outside regarding dog, cat or rabbit, then we also need you to tick "altogether" because we need you to answer how many years of child's life he/she had a dog (regardless of indoor and outdoor). If you have had the same type of animal indoors and outdoors at the same time, do not count this overlapping time in the total time in the altogether row.)

Animal — How old was [child] when he/she first started having the animal at home? (00 if already before the birth of the child) — What was the total time the animal was kept in the home? — indoor / outdoor

☐ Dog indoor |__|__| yr(s), |__|__| mth(s) |__|__| yr(s) |__|__| mth(s)
☐ Dog outdoor |__|__| yr(s), |__|__| mth(s) |__|__| yr(s) |__|__| mth(s)
☐ Dog (altogether) |__|__| yr(s), |__|__| mth(s) |__|__| yr(s) |__|__| mth(s)
☐ Cat indoor |__|__| yr(s), |__|__| mth(s) |__|__| yr(s) |__|__| mth(s)
☐ Cat outdoor |__|__| yr(s), |__|__| mth(s) |__|__| yr(s) |__|__| mth(s)
☐ Cat (altogether) |__|__| yr(s), |__|__| mth(s) |__|__| yr(s) |__|__| mth(s)
☐ Rabbit indoor |__|__| yr(s), |__|__| mth(s) |__|__| yr(s) |__|__| mth(s)
☐ Rabbit outdoor |__|__| yr(s), |__|__| mth(s) |__|__| yr(s) |__|__| mth(s)
☐ Rabbit (altogether) |__|__| yr(s), |__|__| mth(s) |__|__| yr(s) |__|__| mth(s)
☐ Guinea-pig |__|__| yr(s), |__|__| mth(s) |__|__| yr(s) |__|__| mth(s) |__| / |__|
☐ Hamster |__|__| yr(s), |__|__| mth(s) |__|__| yr(s) |__|__| mth(s) |__| / |__|
☐ Mouse/rat |__|__| yr(s), |__|__| mth(s) |__|__| yr(s) |__|__| mth(s) |__| / |__|
☐ Horse/pony |__|__| yr(s), |__|__| mth(s) |__|__| yr(s) |__|__| mth(s)
☐ Pig |__|__| yr(s), |__|__| mth(s) |__|__| yr(s) |__|__| mth(s)
☐ Cow |__|__| yr(s), |__|__| mth(s) |__|__| yr(s) |__|__| mth(s)
☐ Goat |__|__| yr(s), |__|__| mth(s) |__|__| yr(s) |__|__| mth(s)
☐ Sheep |__|__| yr(s), |__|__| mth(s) |__|__| yr(s) |__|__| mth(s)
☐ Donkey |__|__| yr(s), |__|__| mth(s) |__|__| yr(s) |__|__| mth(s)
☐ Other fury animals |__|__| yr(s), |__|__| mth(s) |__|__| yr(s) |__|__| mth(s) |__| / |__|
☐ Chicken, duck, turkey |__|__| yr(s), |__|__| mth(s) |__|__| yr(s) |__|__| mth(s)
☐ Other bird |__|__| yr(s), |__|__| mth(s) |__|__| yr(s) |__|__| mth(s) |__| / |__|
☐ Fish |__|__| yr(s), |__|__| mth(s) |__|__| yr(s) |__|__| mth(s)
☐ Turtle |__|__| yr(s), |__|__| mth(s) |__|__| yr(s) |__|__| mth(s) |__| / |__|
☐ Snakes/reptiles |__|__| yr(s), |__|__| mth(s) |__|__| yr(s) |__|__| mth(s) |__| / |__|
☐ Insects/spiders |__|__| yr(s), |__|__| mth(s) |__|__| yr(s) |__|__| mth(s)
☐ Other animal _________|__|__| yr(s), |__|__| mth(s) |__|__| yr(s) |__|__| mth(s) |__| / |__|
☐ (Do not remember/ do not know)
☐ (No answer)

Flow
I would also like to know whether [child] has had regular contact with animals outside his/her home for a period longer than 6 months – for instance an animal in the day-care centre or in grandparents' or friends' place.

Q 45 Did [child] have regular contact with animals outside his/her home?
If the animal was/is kept at grandparents' or friends' place, please only indicate if [child] spent time there at least 3 times per week.
[Note for the interviewer: Provide interviewees with a list of all the animals.]

☐ Yes
☐ No → *please go to* **Q 47**
☐ (Do not remember/ do not know → *please go to* **Q 47**)
☐ (No answer → *please go to* **Q 47**)

Q 46 What kind of animals/pets? (Show list of animals)
Indoor animal: an animal that is kept inside for most of the time, e.g. sleeps inside, for more than 6 months a year.
(Outdoor animal: an animal that is kept outside for most of the time, for more than 6 months a year.)

(If you tick both inside and outside regarding dog, cat or rabbit, then we also need you to tick "altogether" because we need you to answer how many years of child's life he/she had a dog (regardless of indoor and outdoor). If you have had the same type of animal indoors and outdoors at the same time, do not count this overlapping time in the total time in the altogether row.))

Animal	How old was [child] when he/she first started having contact with the animal? (00 if already before the birth of the child)	What was the total time of contact [child] had with the animal?	indoor / outdoor
☐ Dog indoor	|__|__| yr(s), |__|__| mth(s)	|__|__| yr(s) |__|__| mth(s)	
☐ Dog outdoor	|__|__| yr(s), |__|__| mth(s)	|__|__| yr(s) |__|__| mth(s)	
☐ Dog (altogether)	|__|__| yr(s), |__|__| mth(s)	|__|__| yr(s) |__|__| mth(s)	
☐ Cat indoor	|__|__| yr(s), |__|__| mth(s)	|__|__| yr(s) |__|__| mth(s)	
☐ Cat outdoor	|__|__| yr(s), |__|__| mth(s)	|__|__| yr(s) |__|__| mth(s)	
☐ Cat (altogether)	|__|__| yr(s), |__|__| mth(s)	|__|__| yr(s) |__|__| mth(s)	
☐ Rabbit indoor	|__|__| yr(s), |__|__| mth(s)	|__|__| yr(s) |__|__| mth(s)	
☐ Rabbit outdoor	|__|__| yr(s), |__|__| mth(s)	|__|__| yr(s) |__|__| mth(s)	
☐ Rabbit (altogether)	|__|__| yr(s), |__|__| mth(s)	|__|__| yr(s) |__|__| mth(s)	
☐ Guinea-pig	|__|__| yr(s), |__|__| mth(s)	|__|__| yr(s) |__|__| mth(s)	|__| / |__|
☐ Hamster	|__|__| yr(s), |__|__| mth(s)	|__|__| yr(s) |__|__| mth(s)	|__| / |__|
☐ Mouse/rat	|__|__| yr(s), |__|__| mth(s)	|__|__| yr(s) |__|__| mth(s)	|__| / |__|
☐ Horse/pony	|__|__| yr(s), |__|__| mth(s)	|__|__| yr(s) |__|__| mth(s)	
☐ Pig	|__|__| yr(s), |__|__| mth(s)	|__|__| yr(s) |__|__| mth(s)	
☐ Cow	|__|__| yr(s), |__|__| mth(s)	|__|__| yr(s) |__|__| mth(s)	
☐ Goat	|__|__| yr(s), |__|__| mth(s)	|__|__| yr(s) |__|__| mth(s)	

☐ Sheep	|__|__| yr(s), |__|__| mth(s)	|__|__| yr(s) |__|__| mth(s)	
☐ Donkey	|__|__| yr(s), |__|__| mth(s)	|__|__| yr(s) |__|__| mth(s)	
☐ Other fury animals	|__|__| yr(s), |__|__| mth(s)	|__|__| yr(s) |__|__| mth(s)	|__| / |__|
☐ Chicken, duck, turkey	|__|__| yr(s), |__|__| mth(s)	|__|__| yr(s) |__|__| mth(s)	
☐ Other bird	|__|__| yr(s), |__|__| mth(s)	|__|__| yr(s) |__|__| mth(s)	|__| / |__|
☐ Fish	|__|__| yr(s), |__|__| mth(s)	|__|__| yr(s) |__|__| mth(s)	
☐ Turtle	|__|__| yr(s), |__|__| mth(s)	|__|__| yr(s) |__|__| mth(s)	|__| / |__|
☐ Snakes/reptiles	|__|__| yr(s), |__|__| mth(s)	|__|__| yr(s) |__|__| mth(s)	|__| / |__|
☐ Insects/spiders	|__|__| yr(s), |__|__| mth(s)	|__|__| yr(s) |__|__| mth(s)	
☐ Other animal _________	|__|__| yr(s), |__|__| mth(s)	|__|__| yr(s) |__|__| mth(s)	|__| / |__|
☐ (Do not remember/ do not know)			
☐ (No answer)			

Q 47 Has [child] ever gone horseback riding or spent time in a stable regularly?
In terms of regularly I mean at least once a week for a period of 6 months.

☐ Yes
☐ No → *please go to* **Q 50**
☐ (Do not remember/ do not know → *please go to* **Q 50**)
☐ (No answer → *please go to* **Q 50**)

Q 48 How old was [child] when he/she first started horseback riding or spent time in a stable?

|__|__| years and |__|__| month(s)
☐ (Do not remember/ do not know)
☐ (No answer)

Q 49 What was the total time [child] went horseback riding or spent time in a stable?

|__|__| yr(s) |__|__| mth(s)
☐ (Do not remember/ do not know)
☐ (No answer)

Flow
I would now like to ask you about the animals that mother has been in regular contact with during pregnancy with [child].

Q 50 During the pregnancy were any animals kept at home?
[Note for the interviewer: Provide interviewees with a list of all the animals.]

☐ Yes
☐ No → *please go to* Q 52
☐ (Do not remember/ do not know → *please go to* Q 52)
☐ (No answer → *please go to* Q 52)

Q 51 What kind of animals/pets? (Show list of animals)

Indoor animal: an animal that is kept inside for most of the time, e.g. sleeps inside, for more than 6 months a year.
(Outdoor animal: an animal that is kept outside for most of the time, for more than 6 months a year.)

Animal	indoor / outdoor
☐ Dog	|__| / |__|
☐ Cat	|__| / |__|
☐ Rabbit	|__| / |__|
☐ Guinea-pig	|__| / |__|
☐ Hamster	|__| / |__|
☐ Mouse/rat	|__| / |__|
☐ Horse/pony	
☐ Pig	
☐ Cow	
☐ Goat	
☐ Sheep	
☐ Donkey	
☐ Other furry animals	|__| / |__|
☐ Chicken, duck, turkey	
☐ Other bird	|__| / |__|
☐ Fish	
☐ Turtle	|__| / |__|
☐ Snakes/reptiles	|__| / |__|
☐ Insects/spiders	
☐ Other animal ___________	|__| / |__|
☐ (Do not remember/ do not know)	
☐ (No answer)	

Flow
I would also like to know whether mother has had regular contact with animals outside her home throughout the whole pregnancy.

Q 52 Did mother have regular contact with animals outside her home?
If the animal was kept at parents' or friends' place, please only indicate if mother spent time there at least 3 times per week.
[Note for the interviewer: Provide interviewees with a list of all the animals.]

☐ Yes
☐ No → *please go to* Q 54
☐ (Do not remember/ do not know→ *please go to* Q 54)
☐ (No answer→ *please go to* Q 54)

Q 53 What kind of animals/pets? (Show list of animals)

Indoor animal: an animal that is kept inside for most of the time, e.g. sleeps inside, for more than 6 months a year.
(Outdoor animal: an animal that is kept outside for most of the time, for more than 6 months a year.)

Animal	indoor / outdoor
☐ Dog	|__| / |__|
☐ Cat	|__| / |__|
☐ Rabbit	|__| / |__|
☐ Guinea-pig	|__| / |__|
☐ Hamster	|__| / |__|
☐ Mouse/rat	|__| / |__|
☐ Horse/pony	
☐ Pig	
☐ Cow	
☐ Goat	
☐ Sheep	
☐ Donkey	
☐ Other fury animals	|__| / |__|
☐ Chicken, duck, turkey	
☐ Other bird	|__| / |__|
☐ Fish	
☐ Turtle	|__| / |__|
☐ Snakes/reptiles	|__| / |__|
☐ Insects/spiders	
☐ Other animal ___________	|__| / |__|
☐ (Do not remember/ do not know)	
☐ (No answer)	

Flow
Now, I would like to know about [child]'s and mother's environment during pregnancy and childhood.

Q 54 Did the mother during her pregnancy with [child] primarily live in the city, the countryside or on a farm? (Tick only one)

☐ In a city/town/suburb/village (with 200 inhabitants or more)
☐ On a farm
☐ In the countryside (or in a village with up to 200 inhabitants)
☐ (Do not remember/ do not know)
☐ (No answer)

**Q 55**	**Before [child] turned 6, did he/she primarily live in the city, the countryside or on a farm**? (Tick only one)<br><br>☐ In a city/town/suburb/village (with 200 inhabitants or more)<br>☐ On a farm<br>☐ In the countryside (or in a village with up to 200 inhabitants)<br>☐ (Do not remember/ do not know)<br>☐ (No answer)

**Q 56**	**Who answered these questions?** (Tick only one)<br><br>☐ Child<br>☐ Mother<br>☐ Mother and child<br>☐ Father<br>☐ Father and child<br>☐ Only parents<br>☐ Together

Questions regarding x-rays and scanning

Flow
Now I have some questions about scans and x-rays. (Scans are for instance CT (Computer Tomography), MRI (Magnetic Resonance Imaging), PET (Positron Emission Tomography) or ultrasound.)

**Q 57**	**Has [child] ever had any x-ray or scan examinations performed?** Do not include dental x-rays.<br>(For example caused by suspicion of an illness, a broken bone or the like)<br>(Cases: I would like to hear about examinations that occurred before the diagnosis of the brain tumour.)<br><br>☐ Yes<br>☐ No → *please go to* **Q 65**<br>☐ (Do not remember/ do not know → *please go to* **Q 65**)<br>☐ (No answer → *please go to* **Q 65**)

**Q 58**	**How many times did [child] have an x-ray or scan performed?**<br>If in relation to a broken leg you had 1 examination when it was broken and another 4 weeks after to see if it was healed, then it counts as 2 times.<br><br>|__|__| times

Flow
(If this happened repeatedly, please start with the first time and I will repeat the questions for each time.)

Q 59 What was the reason for this examination?

		(Do not remember/do not know)	(No answer)
1st time	State: ______________________	☐	☐
2nd time	State: ______________________	☐	☐
3rd time	State: ______________________	☐	☐
4th time	State: ______________________	☐	☐

Q 60 What part(s) of his/her body was examined? (Tick only one)
[Note for the interviewer: Categorise the answer without reading aloud the options.]

1st	2nd	3rd	4th	
☐	☐	☐	☐	Head or neck
☐	☐	☐	☐	Thorax (lungs, heart and breast)
☐	☐	☐	☐	Abdomen (stomach)
☐	☐	☐	☐	Legs or arms
☐	☐	☐	☐	Whole body including head or neck
☐	☐	☐	☐	Whole body excluding head or neck
☐	☐	☐	☐	Other: ______________________
☐	☐	☐	☐	(Do not remember/ do not know)
☐	☐	☐	☐	(No answer)

Q 61 At which hospital/clinic was the examination performed?

		(Do not remember/do not know)	(No answer)
1st time	Specify ______________________	☐	☐
2nd time	Specify ______________________	☐	☐
3rd time	Specify ______________________	☐	☐
4th time	Specify ______________________	☐	☐

Q 62 How old was [child] at the time?		(Do not remember/do not know)	(No answer)
1st time	☐ Age: |__|__| years and |__|__| months<br>**or**<br>☐ Date: |__|__| (month) |__|__|__|__| (year)	☐	☐
2nd time	☐ Age: |__|__| years and |__|__| months<br>**or**<br>☐ Date: |__|__| (month) |__|__|__|__| (year)	☐	☐
3rd time	☐ Age: |__|__| years and |__|__| months<br>**or**<br>☐ Date: |__|__| (month) |__|__|__|__| (year)	☐	☐
4th time	☐ Age: |__|__| years and |__|__| months<br>**or**<br>☐ Date: |__|__| (month) |__|__|__|__| (year)	☐	☐

Q 63 Was it an x-ray examination or a sort of scan?

1st	2nd	3rd	4th	
☐	☐	☐	☐	An x-ray examination → *please go to* **Q 59** *(after the last examination go to* **Q 65***)*
☐	☐	☐	☐	A sort of scan
☐	☐	☐	☐	(Do not remember/ do not know → *please go to* **Q 59** *(after the last examination go to* **Q 65***)*)
☐	☐	☐	☐	(No answer → *please go to* **Q 59** *(after the last examination go to* **Q 65***)*)

Q 64 What sort of scan was it? (Tick only one)

1st	2nd	3rd	4th	
☐	☐	☐	☐	CT scan
☐	☐	☐	☐	PET scan
☐	☐	☐	☐	MRI scan
☐	☐	☐	☐	Ultrasound
☐	☐	☐	☐	Other: ______________________________
☐	☐	☐	☐	(Do not remember/ do not know)
☐	☐	☐	☐	(No answer)

(End: Repeated questions Q 59 - Q 64 for each examination)

Q 65 Who answered these questions? (Tick only one)

- ☐ Child
- ☐ Mother
- ☐ Mother and child
- ☐ Father
- ☐ Father and child
- ☐ Only parents
- ☐ Together

Questions regarding the child's health

Q 66 Has [child] ever hit his/her head seriously, for instance, a blow to the head that caused a concussion, a serious wound or a headache?

- ☐ Yes
- ☐ No → *please go to* Q 75
- ☐ (Do not remember/ do not know → *please go to* Q 75
- ☐ (No answer → *please go to* Q 75

Q 67 How many times did [child] hit his/her head seriously?

|__|__| times

Flow

(If this happened repeatedly, please start with the first time and I will repeat the questions for each time.)

Q 68 Did [child] lose consciousness in relation to this?

1st	2nd	3rd	4th	
☐	☐	☐	☐	Yes
☐	☐	☐	☐	No
☐	☐	☐	☐	(Do not remember/ do not know**)**
☐	☐	☐	☐	(No answer)

Q 69 Did it make [child] throw up or feel nauseous?

1st	2nd	3rd	4th	
☐	☐	☐	☐	Yes
☐	☐	☐	☐	No
☐	☐	☐	☐	(Do not remember/ do not know**)**
☐	☐	☐	☐	(No answer)

Q 70	Did [child] get a headache in relation to this?				
	1st	2nd	3rd	4th	
	☐	☐	☐	☐	Yes
	☐	☐	☐	☐	No
	☐	☐	☐	☐	(Do not remember/ do not know)
	☐	☐	☐	☐	(No answer)

Q 71	**How would you characterise the injury? Please choose the explanation that fits best.** (Tick only one box)				
	**The injury caused ....**				
	1st	2nd	3rd	4th	
	☐	☐	☐	☐	internal bleeding
	☐	☐	☐	☐	concussion of the brain
	☐	☐	☐	☐	external bleeding
	☐	☐	☐	☐	a bump, a bruise or swelling
	☐	☐	☐	☐	no visible sign
	☐	☐	☐	☐	(Do not remember/ do not know)
	☐	☐	☐	☐	(No answer)

Q 72	How old was [child] at the time?		(Do not remember/do not know)	(No answer)
	1st time	☐ Age: |__|__| years and |__|__| months<br>**or**<br>☐ Date: |__|__| (month) |__|__|__|__| (year)	☐	☐
	2nd time	☐ Age: |__|__| years and |__|__| months<br>**or**<br>☐ Date: |__|__| (month) |__|__|__|__| (year)	☐	☐
	3rd time	☐ Age: |__|__| years and |__|__| months<br>**or**<br>☐ Date: |__|__| (month) |__|__|__|__| (year)	☐	☐
	4th time	☐ Age: |__|__| years and |__|__| months<br>**or**<br>☐ Date: |__|__| (month) |__|__|__|__| (year)	☐	☐

Q 73	**Did [child] see a doctor in connection with the injury?**
	1st 2nd 3rd 4th
	☐ ☐ ☐ ☐ Yes
	☐ ☐ ☐ ☐ No → *please go to* **Q 68** *(after the last head injury go to* Q 75*)*
	☐ ☐ ☐ ☐ (Do not remember/ do not know → *please go to* **Q 68** *(after the last head injury go to* Q 75*)*)
	☐ ☐ ☐ ☐ (No answer → *please go to* **Q 68** *(after the last head injury go to* Q 75*)*)

Q 74	**Did a doctor say that [child] had concussion of the brain?**
	1st 2nd 3rd 4th
	☐ ☐ ☐ ☐ Yes
	☐ ☐ ☐ ☐ No
	☐ ☐ ☐ ☐ (Do not remember/ do not know)
	☐ ☐ ☐ ☐ (No answer)

(End: Repeated questions Q 68 - Q 74 for each head injury)

Flow

I am also interested in knowing about operations that [child] might have undergone. In the following questions we only want to hear about what happened before [exposure date/month].

Q 75	**Has [child] had his/her tonsils removed?**
	☐ Yes
	☐ No
	☐ (Do not remember/ do not know)
	☐ (No answer**)**

Q 76	**Has [child] had his/her appendix removed?**
	☐ Yes
	☐ No
	☐ (Do not remember/ do not know)
	☐ (No answer**)**

Q 77	**Has [child] ever undergone general anaesthetics?** (During general anaesthetics the child is sleeping. It is often used for instance during operations or in relation to certain examinations.)
	☐ Yes
	☐ No → *please go to* Q 81
	☐ (Do not remember/ do not know → *please go to* Q 81)
	☐ (No answer → *please go to* Q 81**)**

Q 78 How many times did [child] undergo general anaesthetics?

|__|__| times

Flow
(If this happened repeatedly, please start with the first time and I will repeat the questions for each time.)

Q 79 For what kind of operation or examination was this? (Tick only one box)

1st	2nd	3rd	4th	
☐	☐	☐	☐	Appendix operation
☐	☐	☐	☐	Hernia surgery
☐	☐	☐	☐	Insertion of ear drainage tube
☐	☐	☐	☐	Removal of tonsils
☐	☐	☐	☐	Removal of polyps
☐	☐	☐	☐	Bone fracture
☐	☐	☐	☐	Other examination/operation, please specify: ___________________
☐	☐	☐	☐	(Do not remember/ do not know)
☐	☐	☐	☐	(No answer)

Q 80 How old was [child] when he/she was anaesthetised?

		(Do not remember/do not know)	(No answer)
1st time	☐ Age: |__|__| years and |__|__| months<br>**or**<br>☐ Date: |__|__| (month) |__|__|__|__| (year)	☐	☐
2nd time	☐ Age: |__|__| years and |__|__| months<br>**or**<br>☐ Date: |__|__| (month) |__|__|__|__| (year)	☐	☐
3rd time	☐ Age: |__|__| years and |__|__| months<br>**or**<br>☐ Date: |__|__| (month) |__|__|__|__| (year)	☐	☐
4th time	☐ Age: |__|__| years and |__|__| months<br>**or**<br>☐ Date: |__|__| (month) |__|__|__|__| (year)	☐	☐

(End: Repeated questions Q 79 - Q 80 for each time child underwent general anaesthetics)

Q 81 Who answered these questions? (Tick only one)

- ☐ Child
- ☐ Mother
- ☐ Mother and child
- ☐ Father
- ☐ Father and child
- ☐ Only parents
- ☐ Together

The child's allergies

Q 82 Has [child] ever had wheezing or whistling in the chest at any time in the past?

- ☐ Yes
- ☐ No → *please go to* **Q 87**
- ☐ (Do not remember/ do not know → *please go to* **Q 87**)
- ☐ (No answer → *please go to* **Q 87**)

Q 83 If yes, when was the first time [child] had these symptoms?

☐ Child's age at the time: |__|__| years and |__|__| months

or

☐ Date: |__|__| (month) |__|__|__|__| (year)

☐ (Do not remember/ do not know)

☐ (No answer)

Q 84 When did [child] <u>last</u> have any of these symptoms?

☐ Child's age at the time: |__|__| years and |__|__| months

or

☐ Date: |__|__| (month) |__|__|__|__| (year)

☐ (Do not remember/ do not know)

☐ (No answer)

Q 85 In the periods with wheezing, how often, on average, has [child]'s sleep been disturbed due to wheezing?

- ☐ Never woke with wheezing
- ☐ Less than one night per week
- ☐ One or more nights per week
- ☐ (Do not remember/ do not know)
- ☐ (No answer)

Q 86 Has wheezing ever been severe enough to limit [child]'s speech to only one or two words at a time between breaths?

☐ Yes
☐ No
☐ (Do not remember/ do not know)
☐ (No answer)

Q 87 Has [child] ever had asthma?

☐ Yes
☐ No → *please go to* Q 89
☐ (Do not remember/ do not know → *please go to* Q 89)
☐ (No answer → *please go to* Q 89)

Q 88 How do you know that [child] has/had asthma? (Tick more boxes if appropriate)

☐ A doctor told us
☐ Other
☐ (Do not remember/ do not know)
☐ (No answer)

Q 89 Has [child]'s chest sounded wheezy or has [child] had a cough during or after exercise?

☐ Yes
☐ No
☐ (Do not remember/ do not know)
☐ (No answer**)**

Q 90 Has [child] had a dry cough at night, apart from a cough associated with a cold or chest infection?

☐ Yes
☐ No
☐ (Do not remember/ do not know**)**
☐ (No answer)

Q 91 Has there ever been any coughing, wheezing or whistling in the chest in connection with any of the following? (Tick more boxes if appropriate)

- ☐ Cat
- ☐ Dog
- ☐ Grass pollen
- ☐ Birch pollen
- ☐ Other pollen
- ☐ House dust mites
- ☐ Dust
- ☐ Weather related (humidity, coldness, heat)
- ☐ Air pollution (from traffic or tobacco smoke)
- ☐ Strong smells (from paint, perfume, or the like)
- ☐ Other:_________________________________
- ☐ No
- ☐ (Do not remember/ do not know)
- ☐ (No answer)

Q 92 Has [child] ever had problems with sneezing, or a runny or blocked nose when he/she DID NOT have a cold or the flu?

- ☐ Yes
- ☐ No → *please go to* **Q 99**
- ☐ (Do not remember/ do not know → *please go to* **Q 99**)
- ☐ (No answer → *please go to* **Q 99**)

Q 93 Has this nose problem ever been accompanied by itchy-watery eyes?

- ☐ Yes
- ☐ No
- ☐ (Do not remember/ do not know)
- ☐ (No answer)

Q 94 When was the first time [child] had sneezing problems, or a runny or blocked nose, or itchy-watery eyes?

- ☐ Child's age at the time: |__|__| years and |__|__| months

or

- ☐ Date: |__|__| (month) |__|__|__|__| (year)
- ☐ (Do not remember/ do not know)
- ☐ (No answer)

Q 95 When did [child] last have any symptoms (sneezing, or a runny or blocked nose, or red and itchy eyes)?

☐ Child's age at the time: |__|__| years and |__|__| months

or

☐ Date: |__|__| (month) |__|__|__|__| (year)

☐ (Do not remember/ do not know)

☐ (No answer)

Q 96 Have there been any problems with sneezing, or a runny or blocked nose, or red and itchy eyes in connection with any of the following? Do not include problems related to food. (Tick more boxes if appropriate)

☐ Cat

☐ Dog

☐ Birch pollen

☐ Grass pollen

☐ Other pollen

☐ House dust mites

☐ Dust

☐ Air pollution (from traffic or tobacco smoke)

☐ Strong smells (from paint, perfume or the like)

☐ Other:_______________________________

☐ No

☐ (Do not remember/ do not know)

☐ (No answer)

Q 97 In which months of the year did this nose or eye problem normally occur? (Tick as many as appropriate)

January ( ), February ( ), March ( ), April ( ), May ( ),
June ( ), July ( ), August ( ), September ( ), October ( ), November ( ), December ( ),
Any time during the year ( )

☐ (Do not remember/ do not know)

☐ (No answer)

Q 98 How much did this nose problem normally interfere with [child]'s daily activities?

☐ Not at all

☐ A little

☐ A moderate amount

☐ A lot

☐ (Do not remember/ do not know)

☐ (No answer)

Q 99 Has [child] ever had hay fever or allergic rhinitis?

☐ Yes
☐ No → *please go to* **Q 101**
☐ (Do not remember/ do not know → *please go to* **Q 101**)
☐ (No answer → *please go to* **Q 101**)

Q 100 How do you know that [child] has/had allergic rhinitis or "hay fever"? (Tick as many boxes as appropriate)

☐ By skin prick test or a blood sample
☐ A doctor told us
☐ Other
☐ (Do not remember/ do not know)
☐ (No answer)

Q 101 Has [child] ever had an itchy rash which was coming and going for at least 6 months?

☐ Yes
☐ No → *please go to* Q 106
☐ (Do not remember/ do not know → *please go to* Q 106)
☐ (No answer → *please go to* Q 106)

Q 102 Has this itchy rash at any time affected any of the following places: the folds of the elbows, behind the knees, in front of the ankles, under the buttocks, or around the neck, ears or eyes?

☐ Yes
☐ No
☐ (Do not remember/ do not know)
☐ (No answer)

Q 103 When did this itchy rash first occur?

☐ Child's age at the time: |__|__| years and |__|__| months
or
☐ Date: |__|__| (month) |__|__|__|__| (year)
☐ (Do not remember/ do not know)
☐ (No answer)

Q 104 When did [child] <u>last</u> have an itchy rash?

☐ Child's age at the time: |__|__| years and |__|__| months
or
☐ Date: |__|__| (month) |__|__|__|__| (year)
☐ (Do not remember/ do not know)
☐ (No answer)

Q 105 In the periods with an itchy rash, how often, on average, has [child] been kept awake at night by this itchy rash?

☐ Never
☐ Less than one night per week
☐ One or more nights per week
☐ (Do not remember/ do not know)
☐ (No answer**)**

Q 106 Has [child] ever had infantile eczema or atopic dermatitis?

☐ Yes
☐ No → *please go to* Q 108
☐ (Do not remember/ do not know → *please go to* Q 108)
☐ (No answer → *please go to* Q 108)

Q 107 How do you know that [child] has/had eczema? (Tick more boxes if appropriate)

☐ A doctor told us
☐ Other
☐ (Do not remember/ do not know)
☐ (No answer)

Q 108 Who answered these questions? (Tick only one)

☐ Child
☐ Mother
☐ Mother and child
☐ Father
☐ Father and child
☐ Only parents
☐ Together

Illnesses

Flow
Thank you. The remainder of the interview concerns the medical history of [child] and questions regarding his/her parents. [Child] is welcome to stay during the rest of the interview, but can leave at any time if he/she prefers.

Q 109 Did [child] have jaundice during the first month of life? (Jaundice is when the skin becomes yellow. A very frequent condition in newborns.)

☐ Yes
☐ No → *please go to* **Q 111**
☐ (Do not remember/ do not know → *please go to* **Q 111**)
☐ (No answer → *please go to* **Q 111**)

Q 110 Did [child] have light treatment against jaundice?

☐ Yes
☐ No
☐ (Do not remember/ do not know)
☐ (No answer)

Q 111 Was [child] treated for low blood sugar during the first two weeks after birth?
(also called hypoglycaemia)

☐ Yes
☐ No
☐ (Do not remember/ do not know)
☐ (No answer)

Q 112 Did [child] have a fever during the first 12 weeks?

☐ Yes
☐ No
☐ (Do not remember/ do not know)
☐ (No answer)

Q 113 Number of infections in the early years of life

I know it can be difficult to remember when [child] has had different infections when he/she was little. But to make this easier try to think of special events such as end of maternity leave, starting in a new day-care centre, parents' new job, holidays, moving or the birth of a younger sibling. One day of illness = if the child is too ill to attend day-care centre, and/or to maintain daily activities, sleeps and is being cranky instead of playing and eating. If the child is sick for one week during one season, then calculate an average per month and tick "once or less a month". **If the child has been ill in connection with operations or fractures, please do not include this.** [Note for the interviewer: Fill in Appendix 3 – Number of Illnesses. (If they cannot answer per season, ask for the whole year then tick the same frequency in both summer and winter and make a remark that this was done?.)]

Fill in Annex 3 –Number of Illnesses (see separate list).

Flow
The next two questions concern two rare diseases that most people only know if they have been diagnosed with it.

Q 114 Does [child] have Tuberous Sclerosis?

☐ Yes
☐ No
☐ (Do not remember/ do not know)
☐ (No answer)

Q 115 Does [child] have Neurofibromatosis or von Recklinghausen's disease?

☐ Yes
☐ No
☐ (Do not remember/ do not know)
☐ (No answer)

Q 116 Has [child] ever had febrile seizures?

☐ Yes
☐ No → *please go to* **Q 120**
☐ (Do not remember/ do not know → *please go to* **Q 120**)
☐ (No answer → *please go to* **Q 120**)

Q 117 How old was [child] the first time this happened?

☐ Child's age at the time: |__|__| years and |__|__| months
or
☐ Date: |__|__| (month) |__|__|__|__| (year)
☐ (Do not remember/ do not know)
☐ (No answer)

Q 118 How many times has [child] had febrile seizures?

|__|__| attacks

Q 119 How long did the longest seizure last?

☐ Less than 5 minutes
☐ Between 5 minutes and half an hour
☐ More than half an hour
☐ (Do not remember/ do not know)
☐ (No answer)

In the following questions we only want you to mention what happened before [exposure date/month].

Q 120 Has [child] ever had a seizure without having fever?

☐ Yes
☐ No → *please go to* **Q 126**
☐ (Do not remember/ do not know → *please go to* **Q 126**)
☐ (No answer → *please go to* **Q 126**)

Q 121 **How old was [child]?** (If more times, please state the age the first time and I will repeat the questions.)

Child's age: |__|__| years
☐ (Do not remember/ do not know)
☐ (No answer)

Child's age: |__|__| years
☐ (Do not remember/ do not know)
☐ (No answer)

Child's age: |__|__| years
☐ (Do not remember/ do not know)
☐ (No answer)

Q 122 **How many seizures did [child] have when he/she was [age]?**

|__|__| times
☐ (Do not remember/ do not know)
☐ (No answer)

|__|__| times
☐ (Do not remember/ do not know)
☐ (No answer)

|__|__| times
☐ (Do not remember/ do not know)
☐ (No answer)

Q 123 **Did he/she have more seizures?**

☐ Yes→ *please go to* **Q 121**
☐ No
☐ (Do not remember/ do not know)
☐ (NoNo answer)

☐ Yes→ *please go to* **Q 121**
☐ No
☐ (Do not remember/ do not know)
☐ (No answer)

☐ Yes→ *please go to* **Q 121**
☐ No
☐ (Do not remember/ do not know)
☐ (No answer)

Q 124 Has [child] ever had a seizure of 45 minutes or more?

☐ Yes
☐ No → *please go to* Q 125
☐ (Do not remember/ do not know → *please go to* Q 125)
☐ (No answer → *please go to* Q 125)

What was [child]'s age the first time he/she had a seizure of 45 minutes or more?

Child's age: |__|__| years
☐ (Do not remember/ do not know)
☐ (No answer)

Q 125 Has a doctor ever said that [child] has epilepsy?

☐ Yes
☐ No
☐ (Do not remember/ do not know)
☐ (No answer)

Q 126 Who answered these questions? (Tick only one)

☐ Child
☐ Mother
☐ Mother and child
☐ Father
☐ Father and child
☐ Only parents
☐ Together

Milestones in child's development

Q 127 When did [child] first start crawling or getting around?

☐ Age: |__|__| years and |__|__| month(s) → *please go to* **Q 129**
or
☐ Date: |__|__| (month) |__|__|__|__| (year) → *please go to* **Q 129**

☐ (Do not remember/ do not know → *please go to* **Q 128**)
☐ (No answer → *please go to* **Q 128**)

Q 128 If you compare to most other children, was it at about the same age, or unusually early or unusually late?

- ☐ Unusually early
- ☐ About the same age as most other children
- ☐ Unusually late
- ☐ (Do not remember/ do not know)
- ☐ (No answer)

Q 129 When did [child] first start walking without help?

☐ Age: |__|__| years and |__|__| month(s) → *please go to* **Q 131**

or

☐ Date: |__|__| (month) |__|__|__|__| (year) → *please go to* **Q 131**

☐ (Do not remember/ do not know → *please go to* **Q 130**)

☐ (No answer → *please go to* **Q 130**)

Q 130 If you compare to most other children, was it at about the same age, or unusually early or unusually late?

- ☐ Unusually early
- ☐ About the same age as most other children
- ☐ Unusually late
- ☐ (Do not remember/ do not know)
- ☐ (No answer)

Q 131 When did [child] say his/her first word? (I.e. a word that you as a parent understood, such as mum, dad, food, hi.)

☐ Age: |__|__| years and |__|__| month(s) → *please go to* **Q 133**

or

☐ Date: |__|__| (month) |__|__|__|__| (year) → *please go to* **Q 133**

☐ (Do not remember/ do not know → *please go to* **Q 132**)

☐ (No answer → *please go to* **Q 132**)

Q 132 If you compare to most other children, was it at about the same age, or unusually early or unusually late?

- ☐ Unusually early
- ☐ About the same age as most other children
- ☐ Unusually late
- ☐ (Do not remember/ do not know)
- ☐ (No answer)

Q 133 Did you use a baby alarm for [child] in his/her first year of life?

☐ Yes
☐ No → *please go to* Q 136
☐ (Do not remember/ do not know → *please go to* Q 136)
☐ (No answer → *please go to* Q 136**)**

Q 134 How often did you use the baby alarm?

☐ Every day
☐ Several times a week
☐ Once a week or less
☐ (Do not remember/ do not know)
☐ (No answer)

Q 135 Where was the baby alarm typically placed?

☐ In the bed/pram, by the head of the baby (within 25 cm of the head)
☐ In bed/pram, but 25 cm or more away from the child's head
☐ On a table/shelf beside the bed/pram
☐ (Do not remember/ do not know)
☐ (No answer)

Q 136 Who answered these questions? (Tick only one)

☐ Child
☐ Mother
☐ Mother and child
☐ Father
☐ Father and child
☐ Only parents
☐ Together

Questions regarding child's contact with other children

Flow
The following questions concern the people that surround [child]. I am interested in knowing whether [child] had regular contact with other children.

Q 137 Has [child] any biological siblings or biological half-siblings?

☐ Yes
☐ No → *please go to* Q 145
☐ (Do not remember/ do not know → *please go to* Q 145)
☐ (No answer → *please go to* Q 145)

Q 138 How many brothers and sisters (both biological and half-siblings) does [child] have?

|__| siblings or half-siblings

Flow
Now I will repeat some questions for each sibling and half-sibling, starting with the eldest.

Q 139 What is the name of the sibling?

		(Do not remember/do not know)	(No answer)
1st sibling	Name: ________________	☐	☐
2nd sibling	Name: ________________	☐	☐
3rd sibling	Name: ________________	☐	☐
4th sibling	Name: ________________	☐	☐

Q 140 When is his/her birthday?

		(Do not remember/do not know)	(No answer)
1st sibling	Birth date: |__|__| (day) |__|__| (month) |__|__|__|__| (year)	☐	☐
2nd sibling	Birth date: |__|__| (day) |__|__| (month) |__|__|__|__| (year)	☐	☐
3rd sibling	Birth date: |__|__| (day) |__|__| (month) |__|__|__|__| (year)	☐	☐
4th sibling	Birth date: |__|__| (day) |__|__| (month) |__|__|__|__| (year)	☐	☐

Q 141 What was the birth weight of the sibling? (If half-sibling: register the information only for maternal half-siblings)

		(Do not remember/do not know)	(No answer)
1st sibling	Birth weight: |__|__|__|__| gram	☐	☐
2nd sibling	Birth weight: |__|__|__|__| gram	☐	☐
3rd sibling	Birth weight: |__|__|__|__| gram	☐	☐
4th sibling	Birth weight: |__|__|__|__| gram	☐	☐

Q 142 Was the sibling born premature? That is more than 3 weeks before term. (If half-sibling: register the information only for maternal half-siblings)

1st	2nd	3rd	4th	
☐	☐	☐	☐	Yes
☐	☐	☐	☐	No
☐	☐	☐	☐	(Do not remember/ do not know)
☐	☐	☐	☐	(No answer)

Specify in weeks before term!		(Do not remember/do not know)	(No answer)
1st sibling	|__|__| weeks premature	☐	☐
2nd sibling	|__|__| weeks premature	☐	☐
3rd sibling	|__|__| weeks premature	☐	☐
4th sibling	|__|__| weeks premature	☐	☐

Q 143 Does he/she have the same parents as [child]?

1st	2nd	3rd	4th	
☐	☐	☐	☐	Same parents
☐	☐	☐	☐	Same mother
☐	☐	☐	☐	Same father
☐	☐	☐	☐	(Do not remember/ do not know)
☐	☐	☐	☐	(No answer)

Q 144 Did [child] and sibling live apart for at least one year before [child] turned 6?
(Living apart would be if they lived together 8 days or less per month.)

1.	2.	3.	4.	
☐	☐	☐	☐	Yes, the children lived apart
☐	☐	☐	☐	No, the children lived together (also if this sibling is younger than [child]) → *please go to* **Q 145**
☐	☐	☐	☐	(Do not remember/ do not know)
☐	☐	☐	☐	(No answer)

In what period of time did the children live apart?
[Note for the interviewer: Only indicate periods of at least one year.]

1st child From |__|__| (month) |__|__|__|__| (year)
Until |__|__| (month) |__|__|__|__| (year)

☐ (Do not remember/ do not know)
☐ (No answer)

2nd child From |__|__| (month) |__|__|__|__| (year)
Until |__|__| (month) |__|__|__|__| (year)

☐ (Do not remember/ do not know)
☐ (No answer)

3rd child From |__|__| (month) |__|__|__|__| (year)
Until |__|__| (month) |__|__|__|__| (year)
☐ (Do not remember/ do not know)
☐ (No answer)

4th child From |__|__| (month) |__|__|__|__| (year)
Until |__|__| (month) |__|__|__|__| (year)
☐ (Do not remember/ do not know)
☐ (No answer)

[If the children did not live together at all before [child] turned 6, write 77.]

(End: Repeated questions Q 139 - Q 144 for each sibling/half-sibling)

Q 145 Did [child] live with other children before he/she turned 6?
(Other than his/her biological siblings or half-siblings)
☐ Yes
☐ No → *please go to* **Q 149**
☐ (Do not remember/ do not know → *please go to* **Q 149**)
☐ (No answer → *please go to* **Q 149**)

**Q 146**	**How many children did [child] live with before he/she turned 6?**<br>(Apart from the above mentioned biological siblings or half-siblings) |__|__|

Flow
(The following questions will be repeated for each other child, starting with the eldest.)

Q 147 **What is their date of birth?** (or at least approximate birth year)

(Start with the eldest)

		(Do not remember/do not know)	(No answer)
1st child	Birth date: |__|__| (day) |__|__| (month) |__|__|__|__| (year)	☐	☐
2nd child	Birth date: |__|__| (day) |__|__| (month) |__|__|__|__| (year)	☐	☐
3rd child	Birth date: |__|__| (day) |__|__| (month) |__|__|__|__| (year)	☐	☐
4th child	Birth date: |__|__| (day) |__|__| (month) |__|__|__|__| (year)	☐	☐

Q 148 Did [child] and the other child live apart for at least one year before [child] turned 6?
(Living apart would be if they lived together 8 days or less per month.)

1st	2nd	3rd	4th	
☐	☐	☐	☐	Yes, the children lived apart
☐	☐	☐	☐	No, the children lived together (also if other child is younger than [child]) → *please go to* Q 149
☐	☐	☐	☐	(Do not remember/ do not know)
☐	☐	☐	☐	(No answer)

In what period of time did the children live apart?
[Note for the interviewer: Only indicate periods of at least one year.]

1st child From |__|__| (month) |__|__|__|__| (year)
Until |__|__| (month) |__|__|__|__| (year)

☐ (Do not remember/ do not know)
☐ (No answer)

2nd child From |__|__| (month) |__|__|__|__| (year)
Until |__|__| (month) |__|__|__|__| (year)

☐ (Do not remember/ do not know)
☐ (No answer)

3rd child From |__|__| (month) |__|__|__|__| (year)
Until |__|__| (month) |__|__|__|__| (year)

☐ (Do not remember/ do not know)
☐ (No answer)

4th child From |__|__| (month) |__|__|__|__| (year)
Until |__|__| (month) |__|__|__|__| (year)

☐ (Do not remember/ do not know)
☐ (No answer)

[If the children did not live together at all before [child] turned 6, write 77.]

(End: Repeated questions Q 147 - Q 148 for each other child)

Flow
Now I have some questions about the first few years of [child]'s life.

Q 149 Did [child] regularly attend [group] before he/she turned 1?
With regularly I mean once a week or more often. (Tick as many boxes as appropriate)

<table>
<tr><th></th><th></th><th>How many months did [child] attend this?</th><th>How many children were in the group?</th></tr>
<tr><td>Mother & toddler group?</td><td>☐ Yes
☐ No
☐ (Do not remember/do not know)
☐ (No answer)</td><td>☐ |__|__| mth(s)
☐ (Do not remember/do not know)
☐ (No answer)</td><td>☐ <5
☐ 5-10
☐ >10
☐ (Do not remember/do not know)
☐ (No answer)</td></tr>
<tr><td>Play group?</td><td>☐ Yes
☐ No
☐ (Do not remember/do not know)
☐ (No answer)</td><td>☐ |__|__| mth(s)
☐ (Do not remember/do not know)
☐ (No answer)</td><td>☐ <5
☐ 5-10
☐ >10
☐ (Do not remember/do not know)
☐ (No answer)</td></tr>
<tr><td>Gym club?</td><td>☐ Yes
☐ No
☐ (Do not remember/do not know)
☐ (No answer)</td><td>☐ |__|__| mth(s)
☐ (Do not remember/do not know)
☐ (No answer)</td><td>☐ <5
☐ 5-10
☐ >10
☐ (Do not remember/do not know)
☐ (No answer)</td></tr>
<tr><td>Baby swimming?</td><td>☐ Yes
☐ No
☐ (Do not remember/do not know)
☐ (No answer)</td><td>☐ |__|__| mth(s)
☐ (Do not remember/do not know)
☐ (No answer)</td><td></td></tr>
<tr><td>Regular meetings with children of neighbours or friends?</td><td>☐ Yes
☐ No
☐ (Do not remember/do not know)
☐ (No answer)</td><td>☐ |__|__| mth(s)
☐ (Do not remember/do not know)
☐ (No answer)</td><td>☐ <5
☐ 5-10
☐ >10
☐ (Do not remember/do not know)
☐ (No answer)</td></tr>
<tr><td>Other groups?</td><td>☐ Yes
☐ No
☐ (Do not remember/do not</td><td>☐ |__|__| mth(s)
☐ (Do not remember/do not know)</td><td>☐ <5
☐ 5-10
☐ >10</td></tr>
</table>

	know)<br>☐ (No answer)	☐ (No answer)	☐ (Do not remember/do not know)<br>☐ (No answer)

Q 150 Did [child] attend a day-care centre before he/she turned 6?

☐ Yes
☐ No → *please go to* **Q 156**
☐ (Do not remember/ do not know → *please go to* **Q 156**)
☐ (No answer → *please go to* **Q 156**)

Q 151 How many day-care centres did [child] attend before he/she turned 6?

(If [child] attended the same day-care centre during several periods, but separated by a period at home, each period should count separately.)

|__|__| day-care centres

Flow
(Repeat the following questions for each day-care centre.)

Q 152 How old was [child] when he/she was attending the day-care centre?

1st centre
☐ Age: From |__|__| years and |__|__| month(s)
to |__|__| years and |__|__| month(s)

or
☐ Date: From |__|__| (month) |__|__|__|__| (year)
to |__|__| (month) |__|__|__|__| (year)
☐ (Do not remember/ do not know)
☐ (No answer)

2nd centre
☐ Age: From |__|__| years and |__|__| month(s)
to |__|__| years and |__|__| month(s)
or
☐ Date: From |__|__| (month) |__|__|__|__| (year)
to |__|__| (month) |__|__|__|__| (year)
☐ (Do not remember/ do not know)
☐ (No answer)

3rd centre
☐ Age: From |__|__| years and |__|__| month(s)
to |__|__| years and |__|__| month(s)
or
☐ Date: From |__|__| (month) |__|__|__|__| (year)
to |__|__| (month) |__|__|__|__| (year)
☐ (Do not remember/ do not know)
☐ (No answer)

4th centre
☐ Age: From |__|__| years and |__|__| month(s)
to |__|__| years and |__|__| month(s)
or
☐ Date: From |__|__| (month) |__|__|__|__| (year)
to |__|__| (month) |__|__|__|__| (year)
☐ (Do not remember/ do not know)
☐ (No answer)

Q 153 How many children went to this day-care centre, to the same unit as [child]?

1st	2nd	3rd	4th	
☐	☐	☐	☐	<5
☐	☐	☐	☐	5-14
☐	☐	☐	☐	15-24
☐	☐	☐	☐	25 or more
☐	☐	☐	☐	(Do not remember/ do not know)
☐	☐	☐	☐	(No answer)

Q 154	**How many children went to this day-care centre, the entire day-care centre?** [Note for the interviewer: Also ask the question if the day-care facility is very small.]

1st	2nd	3rd	4th	
☐	☐	☐	☐	<15
☐	☐	☐	☐	15-30
☐	☐	☐	☐	31-60
☐	☐	☐	☐	>60
☐	☐	☐	☐	(Do not remember/ do not know)
☐	☐	☐	☐	(No answer)

Q 155	**How many hours a week did [child] spend at the day-care centre?**

1st	2nd	3rd	4th	
☐	☐	☐	☐	Less than 20 hours a week
☐	☐	☐	☐	Between 20 and 35 hours a week
☐	☐	☐	☐	More than 35 hours a week
☐	☐	☐	☐	(Do not remember/ do not know)
☐	☐	☐	☐	(No answer)

(End: Repeated questions Q 152 - Q 155 for each day-care centre)

Q 156 How old was [child] when he/she first started school?

☐ Age: |__|__| years and |__|__| month(s)

or

☐ Date: |__|__| (month) |__|__|__|__| (year)

☐ The child has not started school yet.

☐ (Do not remember/ do not know)

☐ (No answer)

Q 157 Is [child] a “morning” or a “night” person? Is his/her natural daily rhythm to rise early and go to bed early – or late?

[Note for the interviewer: If the child has switched from one to the other, mark “neither”.]

☐ Morning person

☐ Night person

☐ Neither

☐ (Do not remember/ do not know)

☐ (No answer)

Q 158	**Who answered these questions?** (Tick only one)
	☐ Child<br>☐ Mother<br>☐ Mother and child<br>☐ Father<br>☐ Father and child<br>☐ Only parents<br>☐ Together

Questions regarding the child's family's health

Flow
I am also interested in knowing whether any of [child]'s closest biological family members have had certain diseases. By closest relatives I mean mother, father, sister, brother (also including half-siblings, but not step-siblings), grandparents, aunts, uncles and cousins. If aunt or uncle, please specify if it was mother's or father's sister or brother. Also relate cousin and write down the sex.
Has anyone of [child]'s closest biological relatives had any of the following diseases?

Q 159	**Tuberous sclerosis?**
	☐ Yes<br>☐ No<br>☐ (Do not remember/ do not know)<br>☐ (No answer)<br><br>Who: ________________________ (relative's relation to the child)<br><br>Who: ________________________ (relative's relation to the child)

Q 160	**Neurofibromatosis or von Recklinghausen's disease?**
	☐ Yes<br>☐ No<br>☐ (Do not remember/ do not know)<br>☐ (No answer)<br><br>Who: ________________________ (relative's relation to the child)<br><br>Who: ________________________ (relative's relation to the child)

Q 161 Leukaemia?

☐ Yes
☐ No
☐ (Do not remember/ do not know)
☐ (No answer)

Who: ______________________________ (relative's relation to the child)

Who: ______________________________ (relative's relation to the child)

Q 162 Lymphoma?

☐ Yes
☐ No
☐ (Do not remember/ do not know)
☐ (No answer)

Who: ______________________________ (relative's relation to the child)

Who: ______________________________ (relative's relation to the child)

Q 163 Brain tumour?

☐ Yes
☐ No
☐ (Do not remember/ do not know)
☐ (No answer)

Who: ______________________________ (relative's relation to the child)

Who: ______________________________ (relative's relation to the child)

Q 164 Colon cancer?

☐ Yes
☐ No
☐ (Do not remember/ do not know)
☐ (No answer)

Who: ______________________________ (relative's relation to the child)

Who: ______________________________ (relative's relation to the child)

Q 165 Other types of cancer? (For example, breast cancer, skin cancer, lung cancer, ovarian cancer, stomach cancer, cancer of the uterus, cervical cancer, testicular cancer, prostate cancer or others)

☐ Yes
☐ No
☐ (Do not remember/ do not know)
☐ (No answer)

Who: ____________________________ (relative's relation to the child)
Type of cancer: ___________________________

Who: ____________________________ (relative's relation to the child)
Type of cancer: ___________________________

Q 166 Epilepsy?

☐ Yes
☐ No
☐ (Do not remember/ do not know)
☐ (No answer)

Who: ____________________________ (relative's relation to the child)

Who: ____________________________ (relative's relation to the child)

Flow
Note that in the following questions I only ask for parents and siblings, also including half-siblings, but not step-siblings.

Q 167 Has [child]'s biological mother, father or siblings ever had asthma?

☐ Yes
☐ No → *please go to* **Q 171**
☐ (Do not remember/ do not know → *please go to* **Q 171**)
☐ (No answer → *please go to* **Q 171**)

Flow
(Repeat for each relevant person.)

Q 168 Who has had asthma?

☐ 1st - Mother
☐ 2nd - Father
☐ 3rd - Sibling, specify (first name of sibling) ______________________________
☐ 4th - Sibling, specify (first name of sibling) ______________________________
☐ 5th - Sibling, specify (first name of sibling) ______________________________
☐ 6th - Sibling, specify (first name of sibling) ______________________________
☐ (Do not remember/ do not know)
☐ (No answer)

**Q 169**	**Has this been diagnosed by a doctor?**						
	1st	2nd	3rd	4th	5th	6th	
	☐	☐	☐	☐	☐	☐	Yes
	☐	☐	☐	☐	☐	☐	No
	☐	☐	☐	☐	☐	☐	(Do not remember/ do not know)
	☐	☐	☐	☐	☐	☐	(No answer)

**Q 170**	**Are there others who have had asthma?**						
	1st	2nd	3rd	4th	5th	6th	
	☐	☐	☐	☐	☐	☐	Yes → *please go to* Q 168
	☐	☐	☐	☐	☐	☐	No
	☐	☐	☐	☐	☐	☐	(Do not remember/ do not know)
	☐	☐	☐	☐	☐	☐	(No answer)

(End: Repeated questions Q 168 - Q 170
for each person - asthma)

Q 171 Has [child]'s biological mother, father or siblings ever had infantile eczema/atopic dermatitis?

- ☐ Yes
- ☐ No → *please go to* **Q 175**
- ☐ (Do not remember/ do not know → *please go to* **Q 175**)
- ☐ (No answer → *please go to* **Q 175**)

Flow
(Repeat for each relevant person.)

Q 172 Who has had infantile eczema/atopic dermatitis?

- ☐ 1st - Mother
- ☐ 2nd - Father
- ☐ 3rd - Sibling, specify (first name of sibling) ________________________________
- ☐ 4th - Sibling, specify (first name of sibling) ________________________________
- ☐ 5th - Sibling, specify (first name of sibling) ________________________________
- ☐ 6th - Sibling, specify (first name of sibling) ________________________________
- ☐ (Do not remember/ do not know)
- ☐ (No answer)

**Q 173**	**Has this been diagnosed by a doctor?**						
	1st	2nd	3rd	4th	5th	6th	
	☐	☐	☐	☐	☐	☐	Yes
	☐	☐	☐	☐	☐	☐	No
	☐	☐	☐	☐	☐	☐	(Do not remember/ do not know)
	☐	☐	☐	☐	☐	☐	(No answer)

**Q 174**	**Are there others who have had infantile eczema/atopic dermatitis?**						
	1st	2nd	3rd	4th	5th	6th	
	☐	☐	☐	☐	☐	☐	Yes → *please go to* Q 172
	☐	☐	☐	☐	☐	☐	No
	☐	☐	☐	☐	☐	☐	(Do not remember/ do not know)
	☐	☐	☐	☐	☐	☐	(No answer)

(End: Repeated questions Q 172 - Q 174
for each person - eczema)

Q 175 Has the [child]'s biological mother, father or siblings ever had an allergic rhinitis or hay fever? (For example, allergy to pollen or animals with symptoms such as runny nose and itchy and watery eyes.)

☐ Yes
☐ No → *please go to* **Q 179**
☐ (Do not remember/ do not know → *please go to* **Q 179**)
☐ (No answer → *please go to* **Q 179**)

Flow
(Repeat for each relevant person.)

Q 176 Who has had an allergic rhinitis or hay fever?

☐ 1st - Mother
☐ 2nd - Father
☐ 3rd - Sibling, specify (first name of sibling) ______________________________
☐ 4th - Sibling, specify (first name of sibling) ______________________________
☐ 5th - Sibling, specify (first name of sibling) ______________________________
☐ 6th - Sibling, specify (first name of sibling) ______________________________
☐ (Do not remember/ do not know)
☐ (No answer)

**Q 177**	**Has this been diagnosed by a doctor?**						
	1st	2nd	3rd	4th	5th	6th	
	☐	☐	☐	☐	☐	☐	Yes
	☐	☐	☐	☐	☐	☐	No
	☐	☐	☐	☐	☐	☐	(Do not remember/ do not know)
	☐	☐	☐	☐	☐	☐	(No answer)

**Q 178**	**Are there others who have had an allergic rhinitis or hay fever?**						
	1st	2nd	3rd	4th	5th	6th	
	☐	☐	☐	☐	☐	☐	Yes → *please go to* Q 176
	☐	☐	☐	☐	☐	☐	No
	☐	☐	☐	☐	☐	☐	(Do not remember/ do not know)
	☐	☐	☐	☐	☐	☐	(No answer)

(End: Repeated questions Q 176 - Q 178
for each person – hay fever)

Q 179 Who answered these questions? (Tick only one)

☐ Child
☐ Mother
☐ Mother and child
☐ Father
☐ Father and child
☐ Only parents
☐ Together

Questions regarding the pregnancy of the index child

Flow
The rest of the interview are questions that are meant for the mother, but before we continue we would like you to leave a saliva sample.
I now want to ask you some questions about pregnancies and childbirth.

Q 180 Did you work during pregnancy?

☐ Yes
☐ No → *please go to* **Q 189**
☐ (Do not remember/ do not know → *please go to* **Q 189**)
☐ (No answer → *please go to* **Q 189**)

Q 181 Were you at some point during pregnancy off work for more than 3 consecutive weeks before starting your maternity leave?

☐ Yes
☐ No → *please go to* **Q 184**
☐ (Do not remember/ do not know → *please go to* **Q 184**)
☐ (No answer → *please go to* **Q 184**)

Q 182 From which week of pregnancy? (first week off)______________

Until which week of pregnancy? (last week off)_______________
☐ (Do not remember/ do not know)
☐ (No answer)

Q 183 What was the reason for this?

Specify ________________
☐ (Do not remember/ do not know)
☐ (No answer)

**Q 184**	**How many hours per week did you work?**<br><br>☐ Less than 10<br>☐ 10-19<br>☐ 20-29<br>☐ 30-39<br>☐ 40 or more<br>☐ (Do not remember/ do not know)<br>☐ (No answer)

**Q 185**	**Did you work during the daytime, evening, night or did you do shift work?** (Tick only one)<br><br>☐ Daytime<br>☐ Evening<br>☐ Night<br>☐ Shift work, but never during night<br>☐ Shift work, including nights<br>☐ (Do not remember/ do not know)<br>☐ (No answer)

**Q 186**	**What kind of work did you do (title)?**<br><br>Specify ________________________________<br><br>☐ (Do not remember/ do not know)<br>☐ (No answer)<br><br>[Note to interviewer: The job title needs to be specified as precisely as possible, e.g. instead of nurse, write operating nurse.]

**Q 187**	**What were your typical level of activities at work most of the time?** (Tick the one that fits the best)<br><br>☐ Sedentary work<br>☐ Standing work/walking around with the possibility to rest<br>☐ Standing work/walking around without the possibility to rest<br>☐ Lifting jobs<br>☐ (Do not remember/ do not know)<br>☐ (No answer)

**Q 188**	**When did you start your maternity leave? Please state number of weeks before the birth.** (3 days or less = 0 weeks)<br><br>|__|__| weeks<br>☐ (Do not remember/ do not know)<br>☐ (No answer)

Q 189 Did you have or did you develop diabetes during the pregnancy with [child]?

☐ Yes
☐ No → *please go to* **Q 193**
☐ (Do not remember/ do not know → *please go to* **Q 193**)
☐ (No answer → *please go to* **Q 193**)

Q 190 What kind of diabetes?

☐ Maternal diabetes
☐ Normal diabetes (type 1 or type 2)
☐ (Do not remember/ do not know)
☐ (No answer)

Q 191 Did you take insulin during pregnancy in connection with the diabetes?

☐ Yes
☐ No → *please go to* Q 193
☐ (Do not remember/ do not know → *please go to* Q 193)
☐ (No answer → *please go to* Q 193)

Q 192 From which week of pregnancy did you initiate the insulin treatment?
(from start tick 66)

week ___
☐ (Do not remember/ do not know)
☐ (No answer)

Q 193 Did you have any ultrasound examinations performed <u>while</u> pregnant with [child]?
(The result of ultrasound scans is a picture of the foetus shown immediately on a black-and-white television screen.)

☐ Yes
☐ No → *please go to* **Q 196**
☐ (Do not remember/ do not know → *please go to* **Q 196**)
☐ (No answer → *please go to* **Q 196**)

Q 194 How many times did you have ultrasound examinations performed during pregnancy?

|__|__| times

Q 195	In which week of pregnancy did you have the ultrasound examination?	(Do not remember/do not know)	(No answer)
	1st time in ❘__❘__❘ week	☐	☐
	2nd time in ❘__❘__❘ week	☐	☐
	3rd time in ❘__❘__❘ week	☐	☐
	4th time in ❘__❘__❘ week	☐	☐
	5th time in ❘__❘__❘ week	☐	☐

Q 196 **<u>While</u> pregnant with [child], did you have any x-ray or scan examinations performed (apart from UL sound)?** (For example, caused by suspicion of an illness, a broken bone, dental x-ray or the like.) (Scans are for instance CT (Computer Tomography), MRI (Magnetic Resonance Imaging), PET (Positron Emission Tomography).)

- ☐ Yes
- ☐ No → *please go to* **Q 204**
- ☐ (Do not remember/ do not know → *please go to* **Q 204**)
- ☐ (No answer → *please go to* **Q 204**)

Q 197 **How many times did this happen?**

❘__❘__❘ times

Flow

(If this happened repeatedly, please start with the first time and I will repeat the questions for each time.)

Q 198	What was the reason for this examination?		(Do not remember/do not know)	(No answer)
	1st time	State: ________________________	☐	☐
	2nd time	State: ________________________	☐	☐
	3rd time	State: ________________________	☐	☐

Q 199 What part(s) of your body was examined? (Tick only one)
[Note for the interviewer: Categorise the mother's answer without reading the answering options aloud.]

1st	2nd	3rd	
☐	☐	☐	Head or neck
☐	☐	☐	Thorax (lung, heart, breast)
☐	☐	☐	Abdomen (stomach)
☐	☐	☐	Legs or arms
☐	☐	☐	Whole body including head or neck
☐	☐	☐	Whole body excluding head or neck
☐	☐	☐	Dental x-rays
☐	☐	☐	Other ____
☐	☐	☐	(Do not remember/ do not know)
☐	☐	☐	(No answer)

Q 200 How many weeks had you been pregnant at the time of the examination?

		(Do not remember/do not know)	(No answer)
1st time	|__|__| weeks	☐	☐
2nd time	|__|__| weeks	☐	☐
3rd time	|__|__| weeks	☐	☐

Q 201 At which hospital or clinic was the examination performed?

		(Do not remember/do not know)	(No answer)
1st time	Name of hospital/clinic or specify ______________________	☐	☐
2nd time	Name of hospital/clinic or specify ______________________	☐	☐
3rd time	Name of hospital/clinic or specify ______________________	☐	☐

Q 202 Was it an x-ray examination or a sort of scan?

1st	2nd	3rd	
☐	☐	☐	An x-ray examination → *please go to* **Q 198** *(after the last scan go to* **Q 204***)*
☐	☐	☐	A sort of scan
☐	☐	☐	Do not remember/ do not know → *please go to* **Q 198** *(after the last scan go to* **Q 204***)*
☐	☐	☐	**(**No answer→ *please go to* **Q 198** *(after the last scan go to* **Q 204***)*

Q 203 What sort of scan was it? (Tick only one)

1st	2nd	3rd	
☐	☐	☐	CT scan
☐	☐	☐	PET scan
☐	☐	☐	MRI scan
☐	☐	☐	Other:________________________________
☐	☐	☐	(Do not remember/ do not know)
☐	☐	☐	(No answer)

(End: Repeated questions Q 198 - Q 203 for each examination)

Q 204 Did you smoke during pregnancy?

☐ Yes
☐ No
☐ (Do not remember/ do not know)
☐ (No answer)

Q 205 In connection with your pregnancy with [child], did you take any vitamin supplement?

☐ Yes
☐ No → *please go to* **Q 208**
☐ (Do not remember/ do not know → *please go to* **Q 208**)
☐ (No answer → *please go to* **Q 208**)

Q 206 Did you take vitamin supplement containing folic acid?

☐ Yes
☐ No → *please go to* **Q 208**
☐ Do not remember/ do not know → *please go to* **Q 208**)
☐ (No answer → *please go to* **Q 208**)

Q 207 When did you start taking folic acid?

☐ 3 months or more before getting pregnant
☐ Less than 3 months before getting pregnant
☐ After getting pregnant
☐ (Do not remember/ do not know)
☐ (No answer)

Questions regarding the birth

Q 208 What was the birth weight of [child]?

|__|__|__|__| grams
☐ (Do not remember/ do not know)
☐ (No answer)

Q 209 What was the birth length of [child]?

|__|__| cm
☐ (Do not remember/ do not know)
☐ (No answer)

Q 210 When did you give birth compared to your due date? (Tick only one)
(Do not read the options aloud.)

☐ Weeks before _________
☐ Days before _________
☐ Due date
☐ Days after ____________
☐ (Do not remember/ do not know)
☐ (No answer)

Q 211 Is [child] a twin?

☐ Yes
☐ No → please *go to* Q 213
☐ (Do not remember/ do not know → please *go to* Q 213)
☐ (No answer → please *go to* Q 213)

Q 212 Are they monozygotic or dizygotic twins?

☐ Monozygotic
☐ Dizygotic
☐ (Do not remember/ do not know)
☐ (No answer)

Q 213 Was the delivery by caesarean section?

☐ Yes
☐ No
☐ (Do not remember/ do not know)
☐ (No answer)

Q 214 Was a vacuum extractor or forceps used to help the delivery?

☐ Yes, forceps
☐ Yes, vacuum extractor
☐ No
☐ (Do not remember/ do not know)
☐ (No answer)

Q 215 Did you breastfeed [child]?

☐ Yes
☐ No → *please go to* **Q 218**
☐ (Do not remember/ do not know → *please go to* **Q 218**)
☐ (No answer → *please go to* **Q 218**)

Q 216 How many months in total did you breastfeed?
[Note for interviewer: If a period is less than one month, state one month.]

|__|__| months
☐ (Do not remember/ do not know)
☐ (No answer)

Q 217 How many months did you continue breastfeeding [child] when he/she started eating ordinary food? Food with a spoon or bread
[Note for interviewer: If a period is less than one month, state one month. If [child] was not breastfed after the introduction of ordinary food, state 0 months.]

|__|__| months
☐ (Do not remember/ do not know)
☐ (No answer)

Q 218 How much weight did you gain during your pregnancy with [child]?
[Note for interviewer: Kilos gained from the first to the last day of pregnancy, including the weight of the baby]

|__|__| kilo(s)
☐ (Do not remember/ do not know)
☐ (No answer)

Q 219 Have you ever had a miscarriage?

☐ Yes
☐ No → *please go to* **Q 222**
☐ (Do not remember/ do not know → *please go to* **Q 222**)
☐ (No answer → *please go to* **Q 222**)

Q 220 How many miscarriages have you had?

|__|__|

Q 221 In which week of pregnancy did you have the miscarriage(s)?

	Year of Miscarriage	(Do not remember/do not know)	(No answer)
1st time in |__|__| week	|__|__|yyyy	☐	☐
2nd time in |__|__| week	yyyy	☐	☐
3rd time in |__|__| week	yyyy	☐	☐
4th time in |__|__| week	yyyy	☐	☐
5th time in |__|__| week	yyyy	☐	☐

Q 222 Have you ever had an induced abortion because of suspicion of illness or malformation of the foetus?

☐ Yes, **in which year?**

	(Do not remember/ do not know)	(No answer)
1st time in |__|__|__|__|__| year	☐	☐
2nd time in |__|__|__|__|__| year	☐	☐
3rd time in |__|__|__|__|__| year	☐	☐
4th time in |__|__|__|__|__| year	☐	☐
5th time in |__|__|__|__|__| year	☐	☐

☐ No → *please go to* **Q 223**
☐ (Do not remember/ do not know → *please go to* **Q 223**)
☐ (No answer → *please go to* **Q 223**)

Q 223 Have you ever given birth to any stillborn child?

☐ Yes
☐ No → *please go to* **Q 225**
☐ (Do not remember/ do not know → *please go to* **Q 225**)
☐ (No answer → *please go to* **Q 225**)

Q 224 When did it happen?:

1st stillborn birth:

Date: |__|__| (month) |__|__|__|__| (year)

In which week of pregnancy did it happen? : |__|__|
☐ (Do not remember/ do not know)
☐ (No answer)

Did it happen more times?

☐ Yes |__|__| times
☐ No
☐ (Do not remember/ do not know)
☐ (No answer)

2nd stillborn birth:

Date: |__|__| (month) |__|__|__|__| (year)

In which week of pregnancy did it happen? : |__|__|
☐ (Do not remember/ do not know)
☐ (No answer)

More background question, parents

Flow
Finally, I have a few more questions regarding the occupation of the child's parents. The following four questions apply to both the mother and the father. I will talk to the mother first and then the father.

Q 225 From which country is your family originally from? (Biological family)

Mother	Father
☐ (Denmark)	☐ (Denmark)
☐ Other, specify ______________	☐ Other, specify_________________

Q 226 **Please tell me the highest level of formal education you have completed.** [Note for the interviewer: Divorced parents: If child lives with both biological parents alternately: questions refer to the biological parents, not stepparents. If child lives with one stepparent and one biological parent only: the questions refer to the biological parent and the stepparent with whom the child lives. If the child lives with only one parent, fill out the information only for this parent. If child has moved out, note the status before child moved out.]

(country specific!)

Mother/Stepmother	Father/Stepfather	
☐	☐	Primary school
☐	☐	Secondary/high school graduate or less
☐	☐	Graduate of a medium level technical school or professional school
☐	☐	Graduate of university or high level technical school
☐	☐	Postgraduate university
☐	☐	Do not live with this parent
☐	☐	(Do not remember/ do not know)
☐	☐	(No answer)

In the following 2 questions we want you to mention as it was before [exposure date/month].

Q 227 **What is your occupation?** [Note for the interviewer: Divorced parents: If child lives with both biological parents alternately: questions refer to the biological parents, not stepparents. If child lives with one stepparent and one biological parent only: the questions refer to the biological parent and the stepparent with whom the child lives. If the child lives with only one parent, fill out the information only for this parent. If child has moved out, note the status before child moved out.]

(country specific!)

Mother/Stepmother	Father/Stepfather	
☐	☐	Full-time salaried employee
☐	☐	Part-time salaried employee
☐	☐	Self-employed
☐	☐	Freelancer
☐	☐	Student, trainee or apprentice
☐	☐	Employed with salary subsidy in the private or public sector
☐	☐	Unemployed
☐	☐	Taking leave of absence
☐	☐	Housewife/househusband
☐	☐	On sick leave
☐	☐	Earlier retirement benefit
☐	☐	Pensioner
☐	☐	Other
☐	☐	Do not live with this parent
☐	☐	(Do not remember/ do not know)
☐	☐	(No answer)

Q 228 **What is your occupational title?** (If not employed, please state latest occupational title.) [Note for the interviewer: Divorced parents: If child lives with both biological parents alternately: questions refer to the biological parents, not stepparents. If child lives with one stepparent and one biological parent only: the questions refer to the biological parent and the stepparent with whom the child lives. If the child lives with only one parent, fill out the information only for this parent. If child has moved out, note the status before child moved out.]

(country specific!)

Mother/Stepmother	Father/Stepfather	
☐	☐	Top management in companies and the public sector
☐	☐	University graduate (e.g. doctor, lawyer, journalist)
☐	☐	Work with intermediate level skills (e.g. optician, nurse, senior officer in a bank or post office)
☐	☐	Office worker (e.g. bank clerk, secretary)
☐	☐	Sales worker, service worker and care worker (e.g. waiter, hairdresser, police(wo)man, salesman)
☐	☐	Skilled work in agriculture, gardening, forestry, hunting or fishing
☐	☐	Tradesman (e.g. bicycle repairer, painter, mechanic, carpenter)
☐	☐	Other work (e.g. cleaner, kitchen assistant, delivery man)
☐	☐	Military work
☐	☐	Do not live with this parent
☐	☐	(Do not remember/ do not know)
☐	☐	(No answer)

Q 229 **What are mother's and father's birth dates?**

Mother |__|__| (day) |__|__| (month) |__|__|__|__| (year)
Father |__|__| (day) |__|__| (month) |__|__|__|__| (year)
☐ (Do not remember/ do not know)
☐ (No answer)

Q 230 **End of interview**

_ _ : _ _ (hours, minutes)

Q 231 **Comments from the interviewees?**

Flow
Lastly, I would like to ask you the following.

Q 232 Can we contact you at a later time if we are in need of further information for the study?

☐ Yes
☐ No
☐ (Do not remember/ do not know)
☐ (No answer)

Q 233 Would you be interested in receiving a newsletter telling about the results of the study?
(This will not be available until at least a couple of years from now.)

☐ Yes
☐ No
☐ (Do not remember/ do not know)
☐ (No answer)

Flow
Thank you very much for your time.

Q 234 What is the number written on the saliva?

Number |__|__|__|__|__|__|__|__|

Q 235 Was the child health record used at the interview?

☐ Yes
☐ No

Flow
Evaluation of interview by the interviewer

How did you find the quality of the contact with you and the family?

	Parents	Child
☐ Very good	☐	☐
☐ Good	☐	☐
☐ Fairly good	☐	☐
☐ Bad	☐	☐

General skills in recalling

	Parents	Child
Very good......................................	☐	☐
Good..	☐	☐
Fairly good	☐	☐
Bad	☐	☐

Do you think that the questions regarding the following subjects were trustworthy?

Yes	No	Doubtful	
☐	☐	☐	Smoking
☐	☐	☐	Mobil phone
☐	☐	☐	Early infections

Other comments that might influence the quality of the answers: -------------------------------------
----

